# Supplementary material for: Mechanically robust supramolecular polymer co-assemblies
Source: Nat Commun. 2022 Jan 18;13:356. doi: 10.1038/s41467-022-28017-0 (PMC8766479; doi:10.1038/s41467-022-28017-0)
Supplement: Supplementary file 1 — Supplementary Information [file 41467_2022_28017_MOESM1_ESM.pdf]

SUPPLEMENTARY INFORMATION

Mechanically robust supramolecular polymer co-assemblies

Julien Sautaux[1], Franziska Marx[1], Ilja Gunkel[1], Christoph Weder[,1], Stephen Schrettl*[,1]*

[1] Adolphe Merkle Institute, University of Fribourg, Chemin des Verdiers 4, CH-1700 Fribourg, Switzerland

Email: christoph.weder@unifr.ch; stephen.schrettl@unifr.ch

Table of Contents

1. **Supplementary Figures 1−38** **3**
2. **Supplementary Tables 1−11** **41**
3. **Supplementary Methods 1** **52**
4. **Supplementary Methods 2** **56**
5. **Supplementary Methods 3** **59**
6. **Supplementary References** **62**

1. Supplementary Figures 1−38

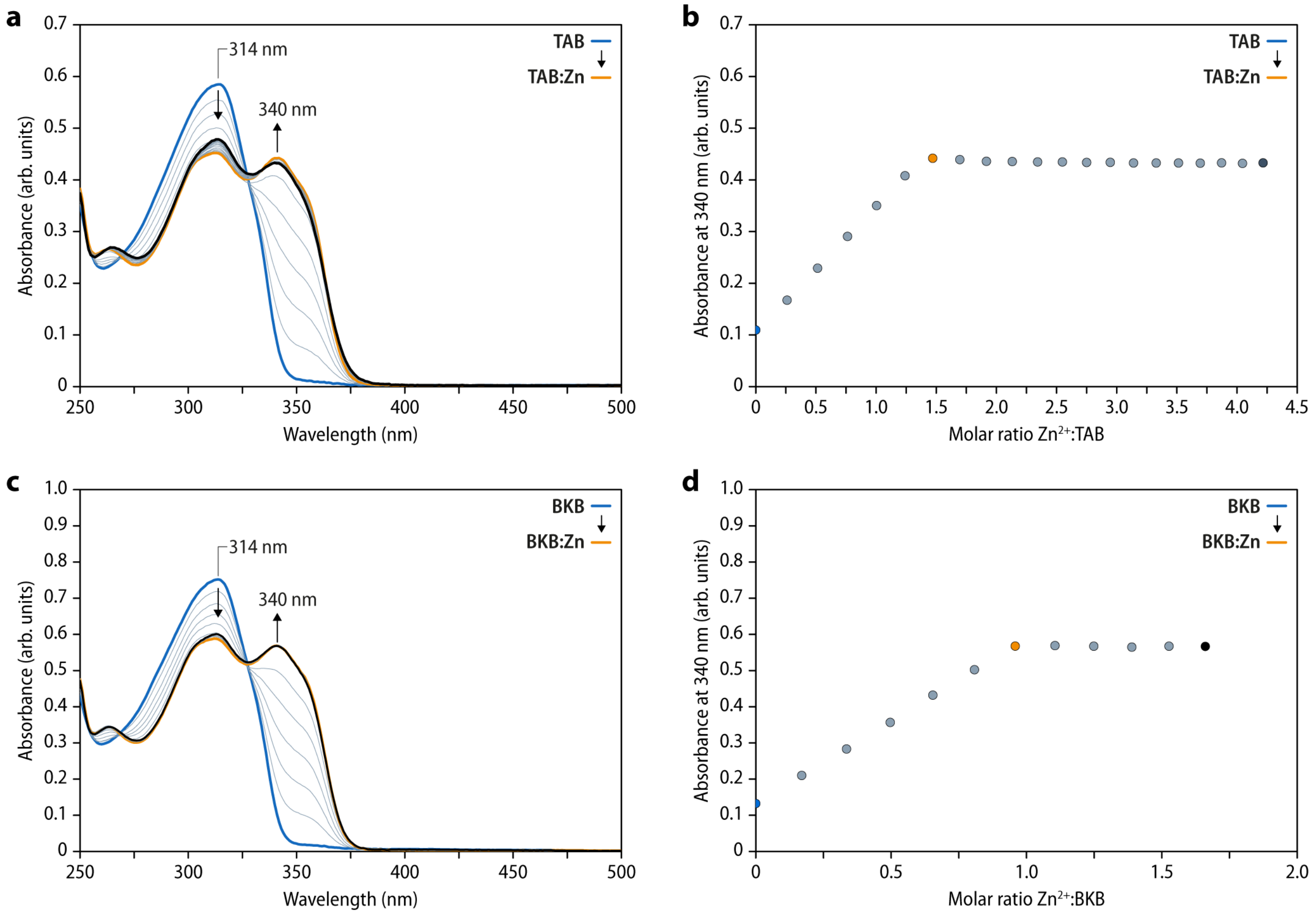

Supplementary Figure 1. a) UV-vis absorption spectrum of a solution of TAB (c = 6.0 µmol L^{-1} in $CHCl_3/CH_3CN$ 9:1 (v/v), blue line) and the spectra of the same solution recorded upon titration with 25 µL aliquots of a solution of $Zn(NTf_2)_2$ (c = 126 µmol L^{-1}) with TAB (c = 6.0 µmol L^{-1}) in $CHCl_3/CH_3CN$ (9:1 v/v). The formation of the coordination complex is observed (thin grey lines) and the spectra no longer change at a metal-to-ligand ratio of 1:2 (orange line). **b)** A plot of the absorbance at 340 nm as a function of the Zn^{2+}:TAB ratio. **c)** UV-vis absorption spectra of a solution of BKB (c = 9.4 µmol L^{-1} in $CHCl_3/CH_3CN$ 9:1 (v/v), blue line) and the spectra of the same solution recorded upon titration with 25 µL aliquots of a solution of $Zn(NTf_2)_2$ (c = 129 µmol L^{-1}) and BKB (c = 9.4 µmol L^{-1}) in a $CHCl_3/CH_3CN$ (9:1 v/v), in which case the formation of the coordination complex is observed (thin grey lines) until the spectra no longer change at a metal-to-ligand ratio of 1:2 (orange line). **d)** A plot of the absorbance at 340 nm as a function of the Zn^{2+}: BKB ratio.

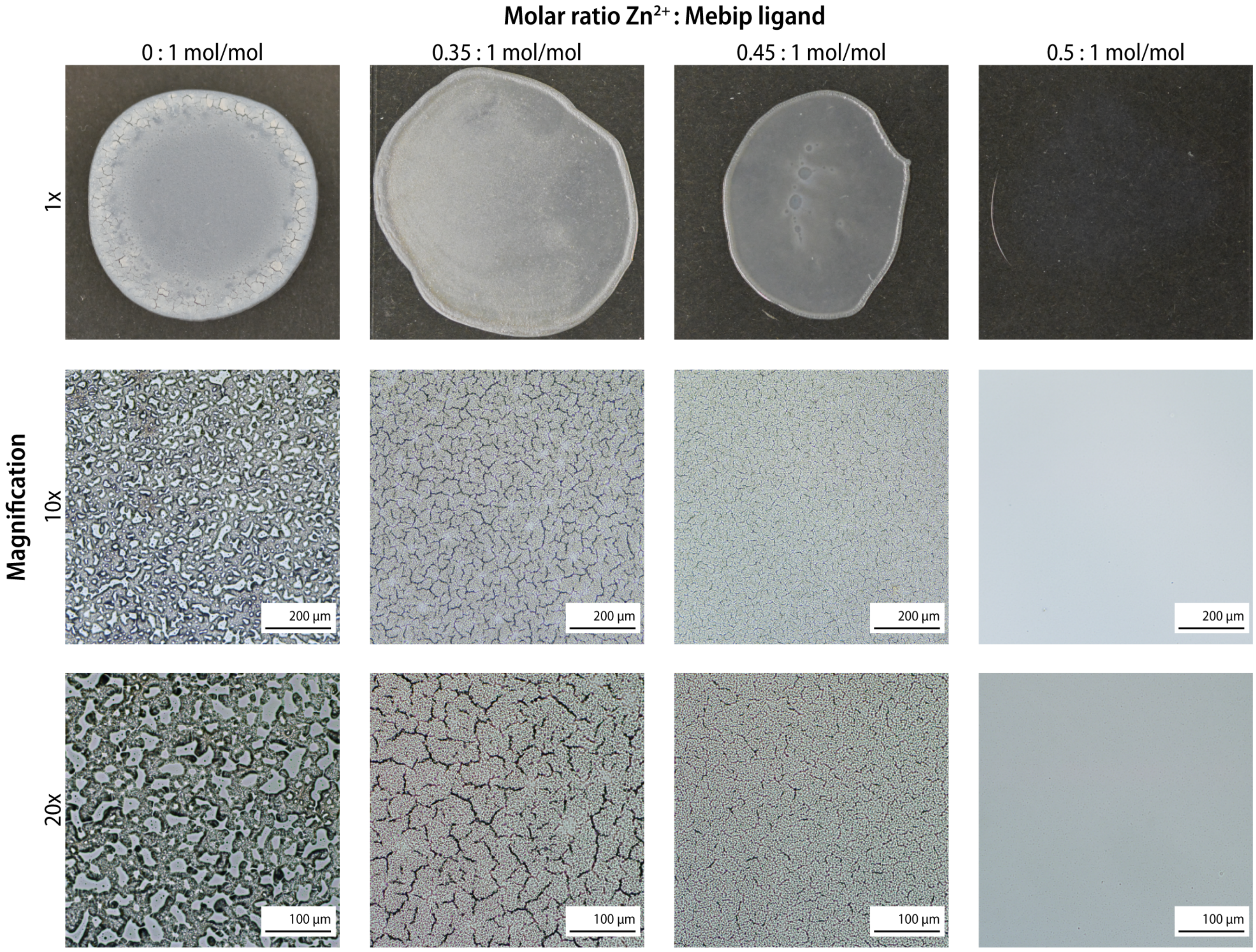

Supplementary Figure 2. Photographs (top row, magnification = 1x) and optical micrographs (middle and bottom rows, magnification = 10x and 20x) of TAB/BKB:Zn films containing TAB and BKB in a 50:50 wt/wt% ratio. The Zn^{2+}:Mebip ratio was varied from 0:1 to 0.5:1 mol/mol. All films were prepared from stirred solutions of TAB and BKB (50:50 wt/wt%) in $CHCl_3$ to which a solution of $Zn(NTf_2)_2$ in anhydrous CH_3CN was added dropwise to adjust the indicated molar Zn^{2+}:Mebip ratios. The mixtures were stirred for 30 min and cast onto quartz glass slides, which were placed in a vacuum oven at 50 °C for 1 day.

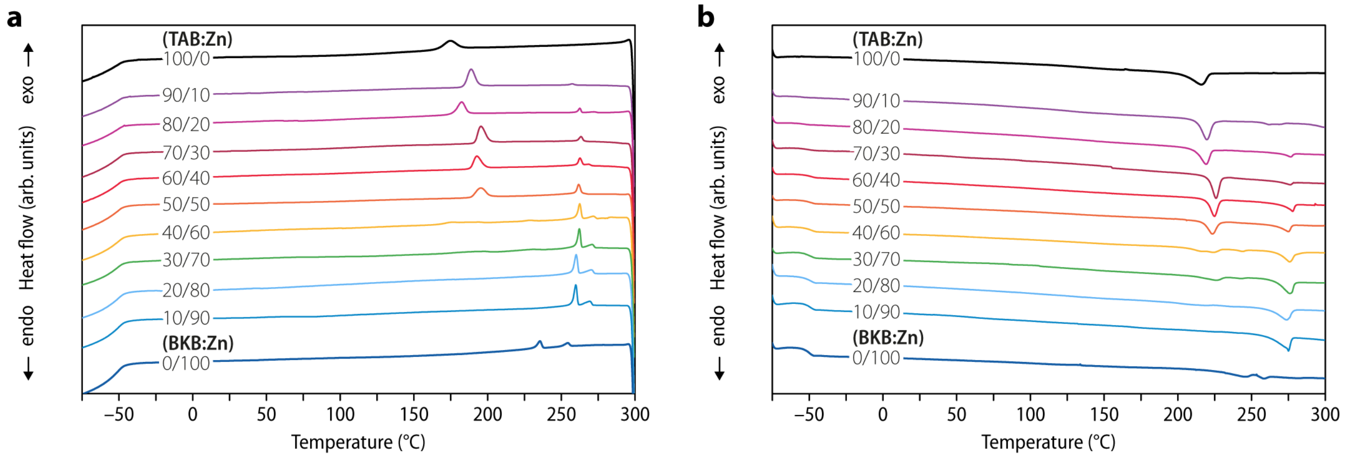

Supplementary Figure 3. Differential scanning calorimetry (DSC) traces showing **(a)** the first cooling scans and **(b)** the second heating scans of the neat metallosupramolecular polymers TAB:Zn and BKB:Zn, as well as of the TAB/BKB:Zn copolymers with the indicated TAB/BKB weight ratio (wt/wt%). The experiments were carried out with heating and cooling rates of 10 °C min^{-1}. The DSC traces are vertically shifted for clarity.

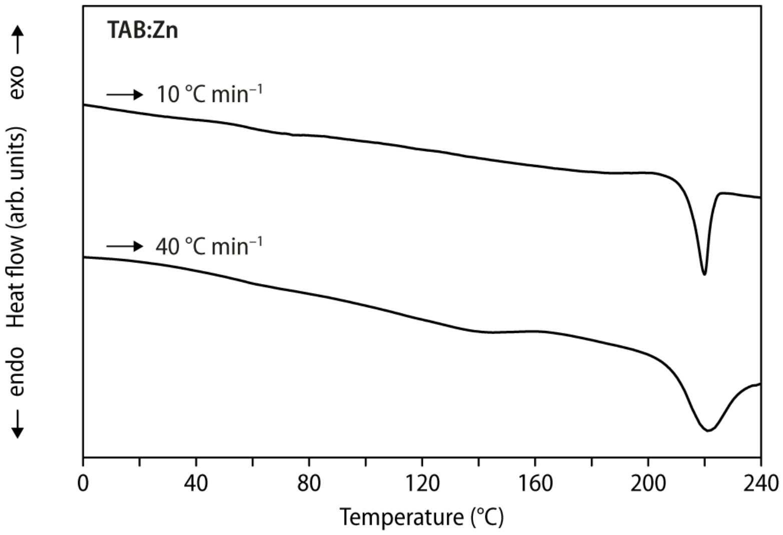

Supplementary Figure 4. Differential scanning calorimetry (DSC) traces showing the first heating scan of a solution-cast TAB:Zn film that was measured with a heating rate of 10 °C min^{-1} (top) and 40 °C min^{-1} (bottom). When the experiment was carried out with the higher heating rate, the DSC traces of TAB:Zn show a glass transition temperature (T_g) at ca. 140 °C.

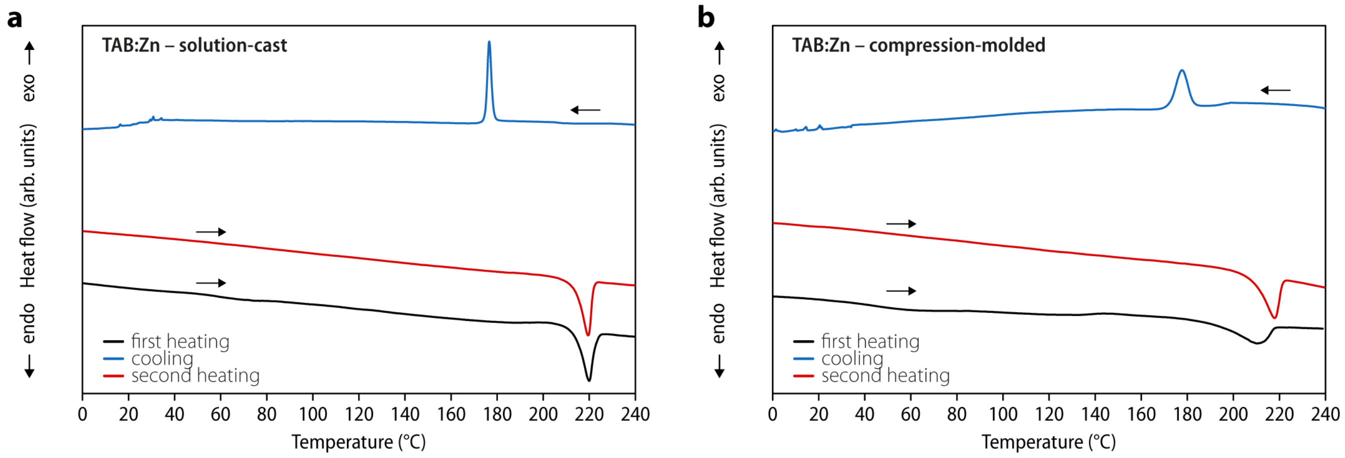

Supplementary Figure 5. Differential scanning calorimetry (DSC) traces showing the first and second heating and the first cooling scan of **(a)** a solution-cast film of TAB:Zn and **(b)** a compression-molded film of TAB:Zn. The experiments were carried out with heating and cooling rates of 10 °C min^{-1}. The DSC traces are vertically shifted for clarity.

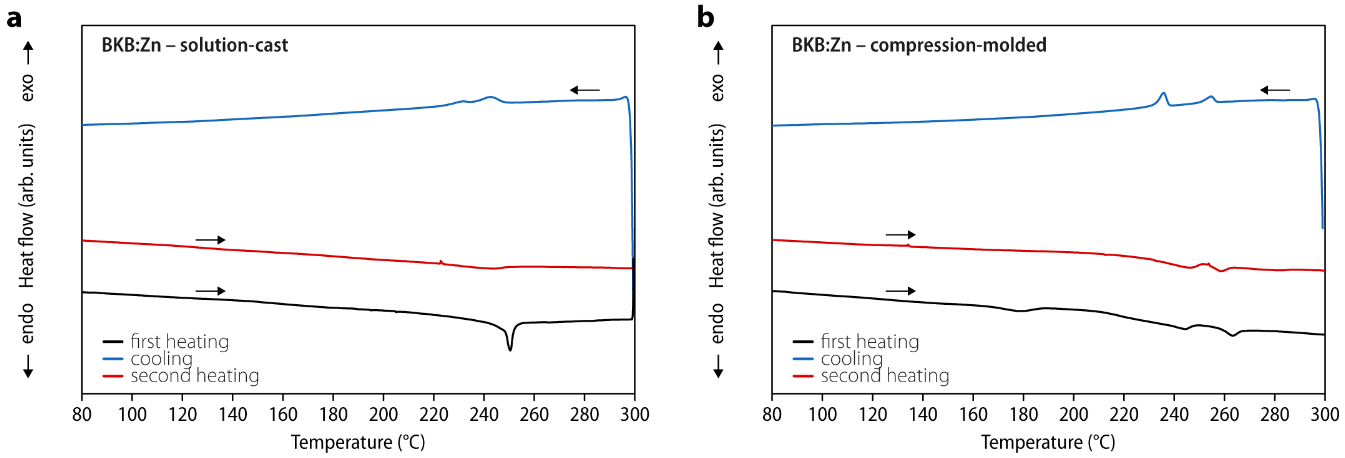

Supplementary Figure 6. Differential scanning calorimetry (DSC) traces showing the first and second heating and the first cooling scan of **(a)** a solution-cast film of BKB:Zn and **(b)** a compression-molded film of BKB:Zn. The experiments were carried out with heating and cooling rates of 10 °C min^{-1}. The DSC traces are vertically shifted for clarity.

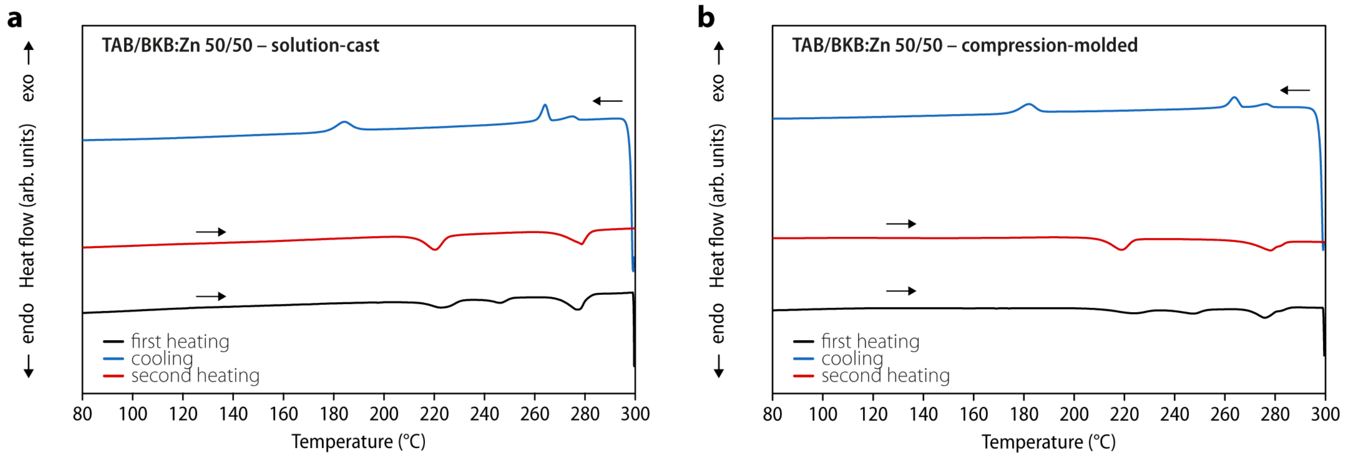

Supplementary Figure 7. Differential scanning calorimetry (DSC) traces showing the first and second heating and the first cooling scan of **(a)** a solution-cast film of TAB/BKB:Zn 50/50 and **(b)** a compression-molded film of TAB/BKB:Zn 50/50. The experiments were carried out with heating and cooling rates of 10 °C min^{-1}. The DSC traces are vertically shifted for clarity.

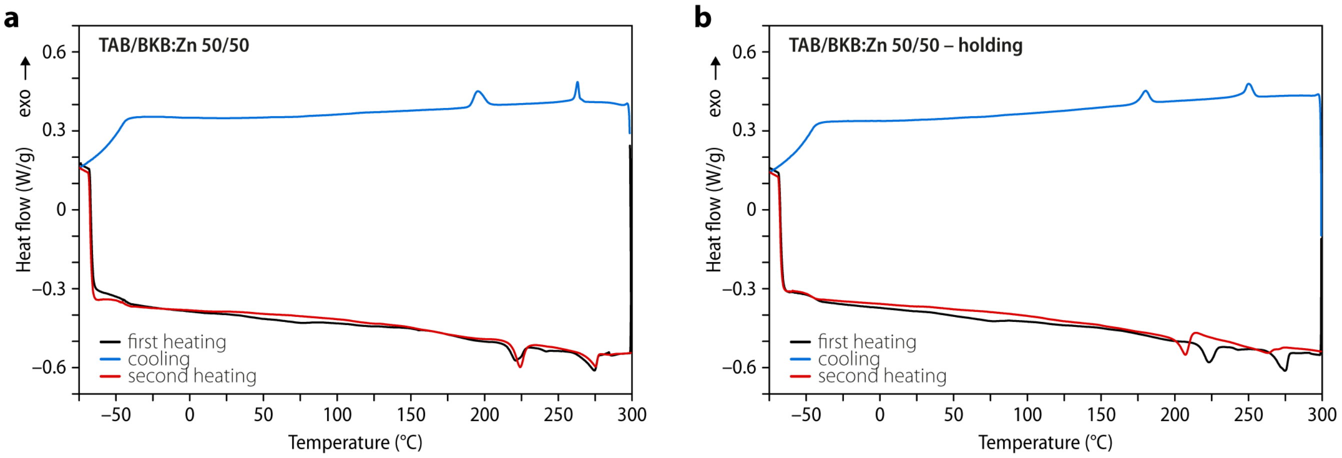

Supplementary Figure 8. Differential scanning calorimetry (DSC) traces showing the first and second heating and the first cooling scan of **(a)** a compression-molded film of TAB/BKB:Zn 50/50 and **(b)** the scans for a compression-molded film of TAB/BKB:Zn 50/50 that was held isothermally at 300 °C for 10 min after the first heating scan. The experiments were carried out with heating and cooling rates of 10 °C min^{-1}.

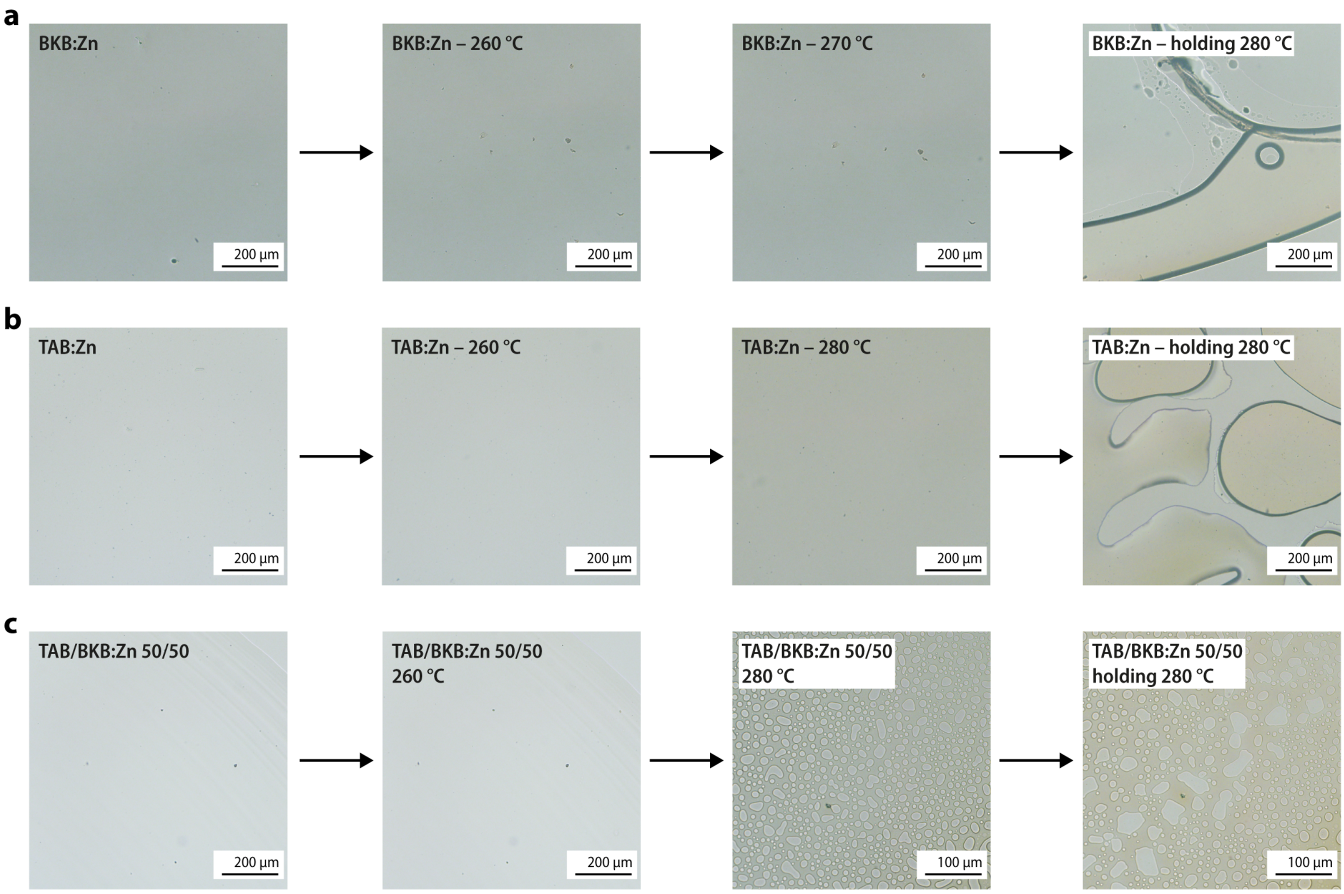

Supplementary Figure 9. Optical micrographs of a solvent-cast films of **(a)** BKB:Zn, **b)** TAB:Zn, and **(c)** TAB/BKB:Zn 50/50 recorded at room temperature (left) and at the indicated temperatures. Prolonged heating to 280 °C under ambient conditions is observed to degrade samples, as indicated by a discoloration when this high temperature is maintained for 10 min (right). The microscopy experiments were carried out with heating and cooling rates of 10 °C min^{-1}.

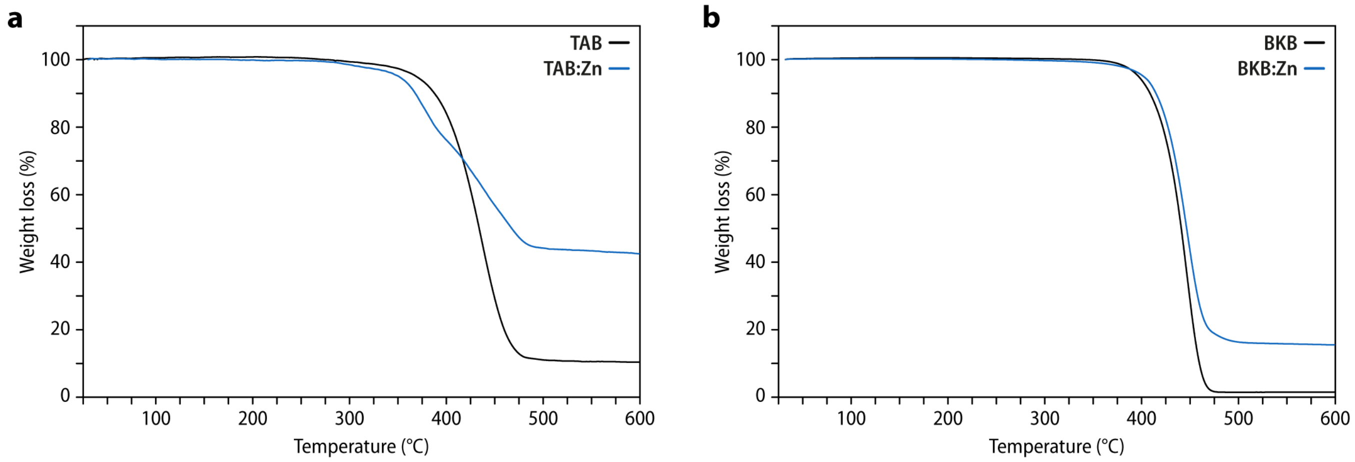

Supplementary Figure 10. Thermogravimetric analyses traces of **(a)** TAB and TAB:Zn as well as **(b)** BKB and BKB:Zn recorded under a nitrogen atmosphere with a heating rate of 10 °C min^{-1}. The TGA measurements show a weight loss of 5 wt% at temperatures of 369 °C for TAB, 351 °C for TAB:Zn, 397 °C for BKB, and 402 °C for BKB:Zn. These findings indicate that substantial thermal degradation of the (macro)monomers and metallosupramolecular polymers only occurs at temperatures above 300 °C.

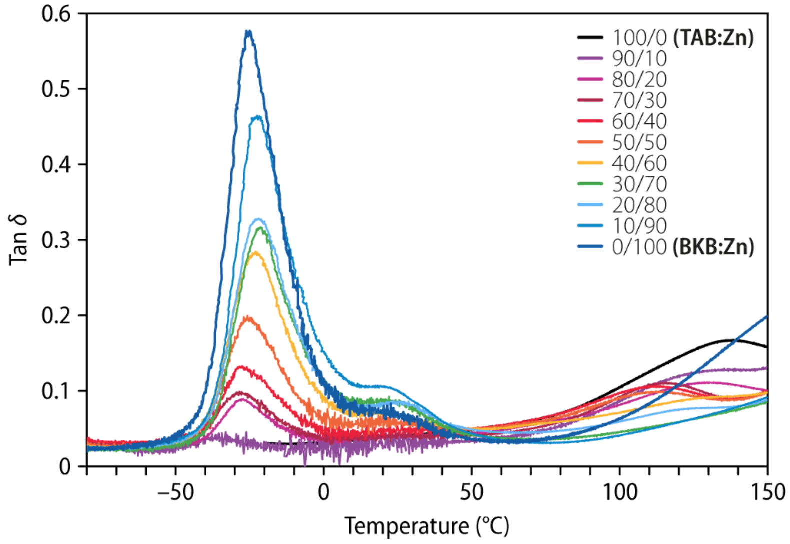

Supplementary Figure 11. Tan *δ* curves acquired by dynamic mechanical analyses (DMA) of the neat metallosupramolecular polymers TAB:Zn and BKB:Zn, as well as of the TAB/BKB:Zn copolymers with the indicated TAB/BKB weight ratio (wt/wt%).

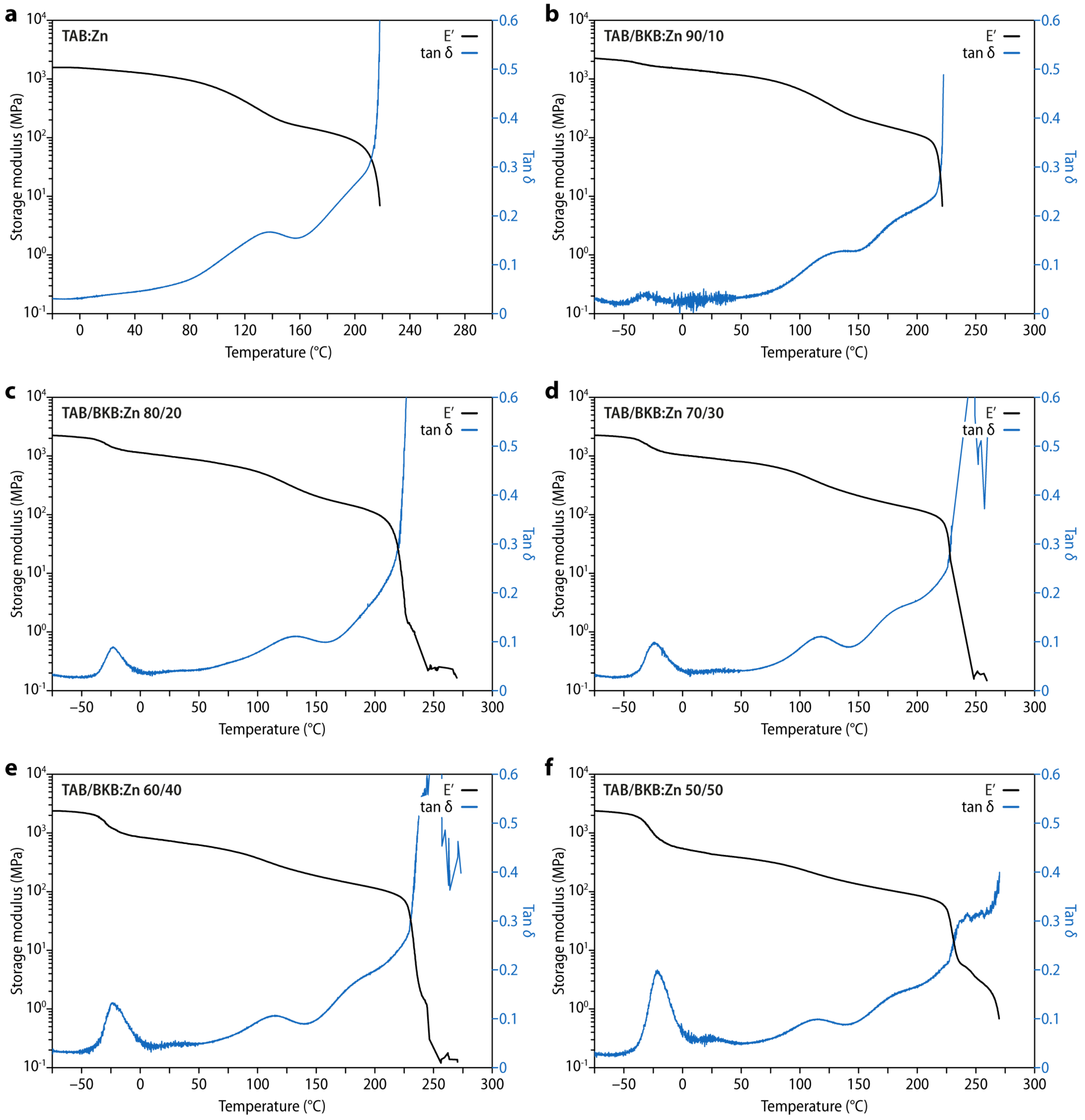

Supplementary Figure 12. Dynamic mechanical analysis (DMA) traces of **(a)** the neat metallosupramolecular polymer TAB:Zn and of **(b–f)** the TAB/BKB:Zn copolymers with the indicated TAB/BKB weight ratio between 90/10 and 50/50 wt/wt%. Shown are the storage modulus (black) and the tan δ (blue) traces.

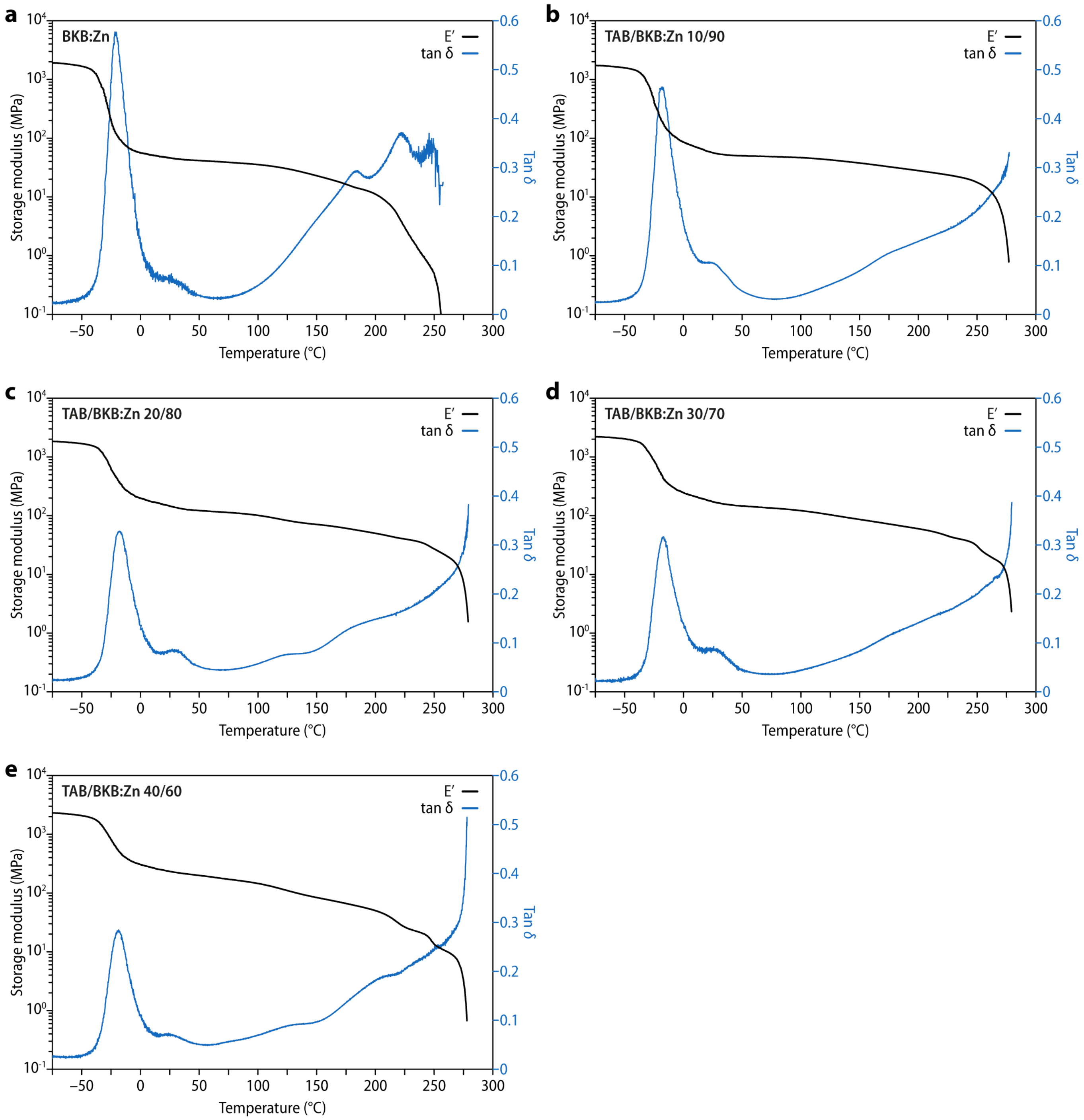

Supplementary Figure 13. Dynamic mechanical analysis (DMA) traces of **(a)** the neat metallosupramolecular polymer BKB:Zn and of **(b–e)** the TAB/BKB:Zn copolymers with the indicated TAB/BKB weight ratio between 10/90 and 40/60 wt/wt%. Shown are the storage modulus (black) and the tan δ (blue) traces.

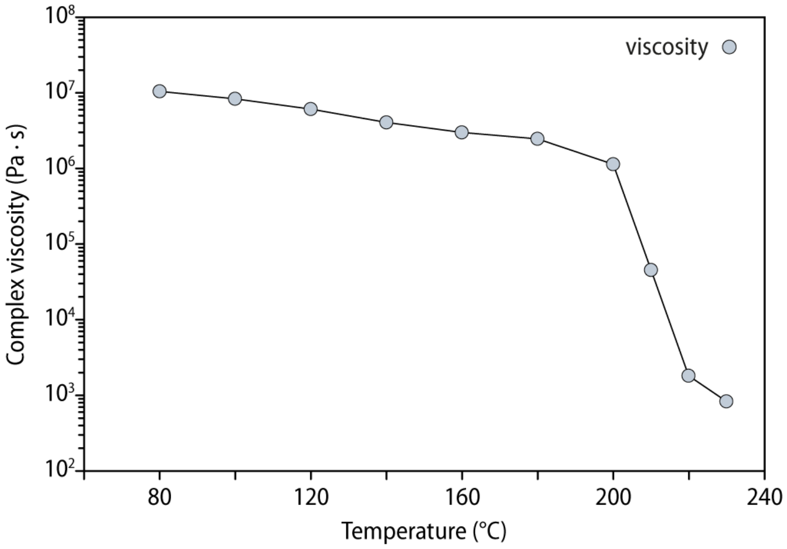

Supplementary Figure 14. Plot of the complex viscosity of a sample of TAB:Zn as a function of temperature as measured with a temperature-controlled parallel plate setup in a rheometer. A gradual viscosity decrease is observed upon heating to 200 °C, corroborating a softening with increasing temperature and mirroring the behavior observed by DMA. Upon further heating, TAB:Zn undergoes a substantial reduction in viscosity from 1.13 10^6 Pa s at 200 °C to only 827 Pa s at 230 °C, characteristic of a solid-to-fluid transition that confirms the melting of the MSP network. Data were recorded at 25 rad/s.

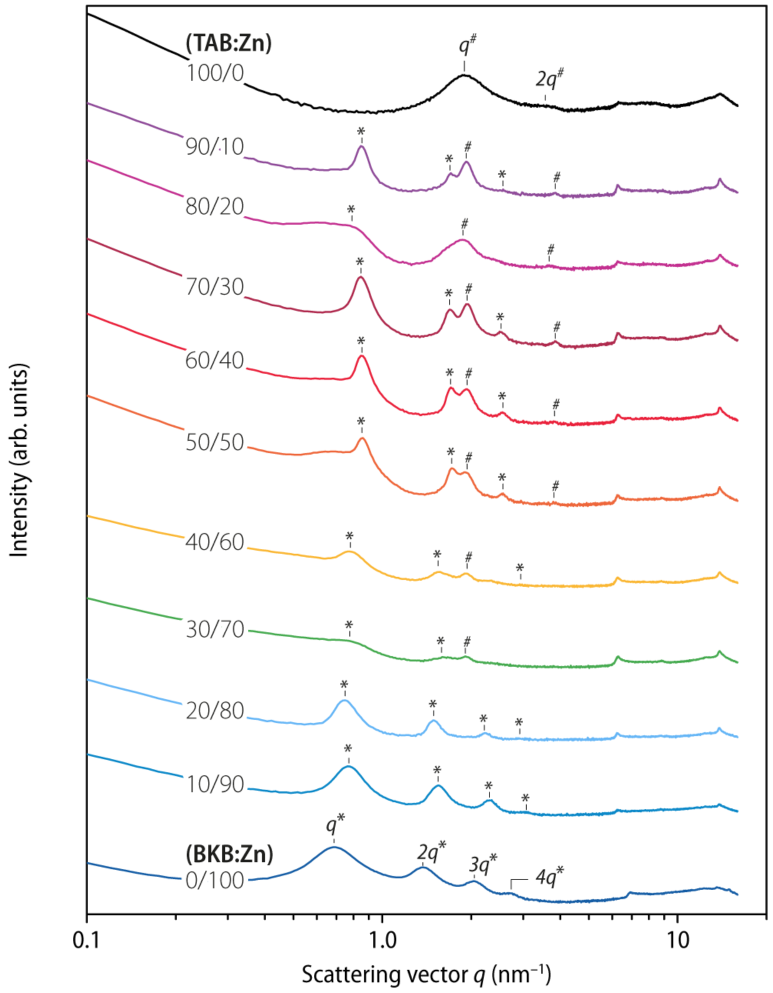

Supplementary Figure 15. Small- and wide-angle X-ray scattering (SAXS/WAXS) profiles of the neat metallosupramolecular polymers TAB:Zn and BKB:Zn, and the TAB/BKB:Zn copolymers with the indicated TAB/BKB weight ratio (wt/wt%). Scattering profiles are shifted vertically for clarity.

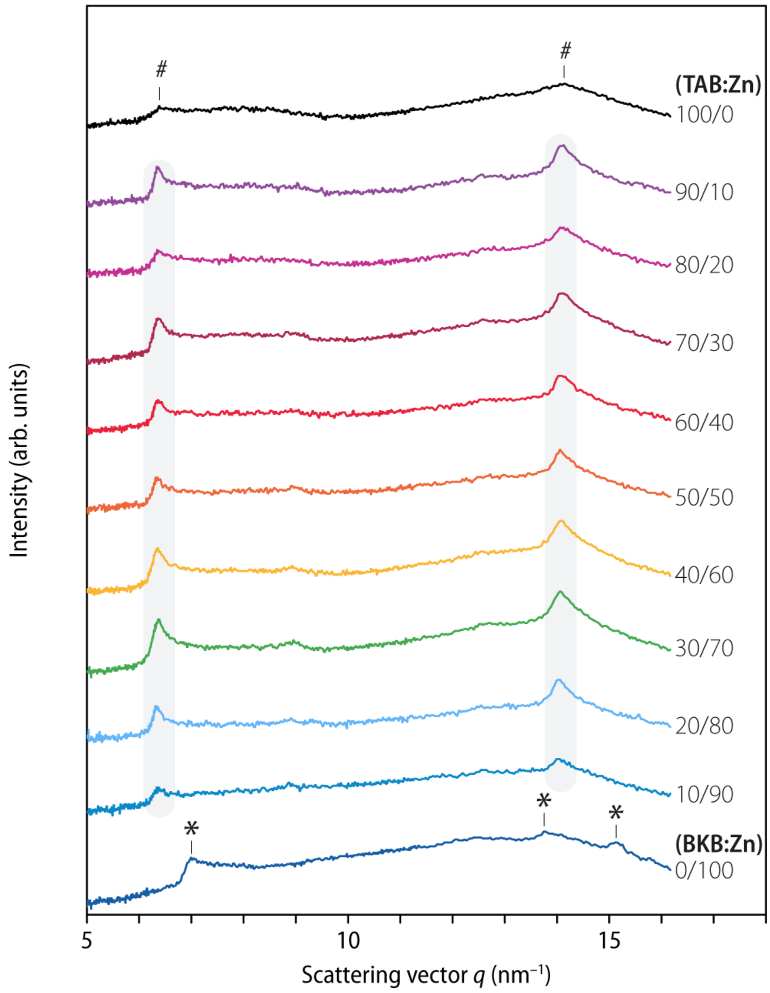

Supplementary Figure 16. Wide-angle X-ray scattering (WAXS) profiles of the neat metallosupramolecular polymers TAB:Zn and BKB:Zn, and the TAB/BKB:Zn copolymers with the indicated TAB/BKB weight ratio (wt/wt%). Scattering profiles are shifted vertically for clarity.

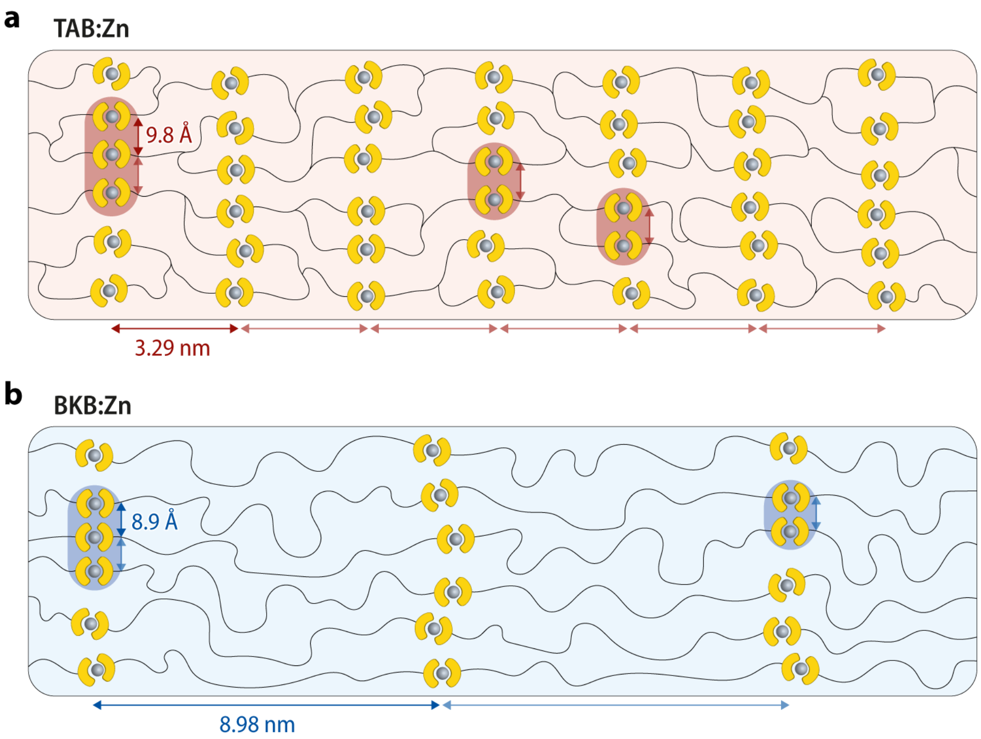

Supplementary Figure 17. Schematic representation of the possible morphologies of the neat metallosupramolecular polymers **(a)** TAB:Zn and **(b)** BKB:Zn. The indicated characteristic spacings were determined by analysis of the small- and wide-angle X-ray scattering (SAXS/WAXS) profiles (see Supplementary Figures 15-16 and Supplementary Table 2). The WAXS profiles show characteristic spacings that indicate the presence of crystalline domains of the metal ligand complexes in the neat metallosupramolecular polymers, which are highlighted in dark red and blue, respectively.

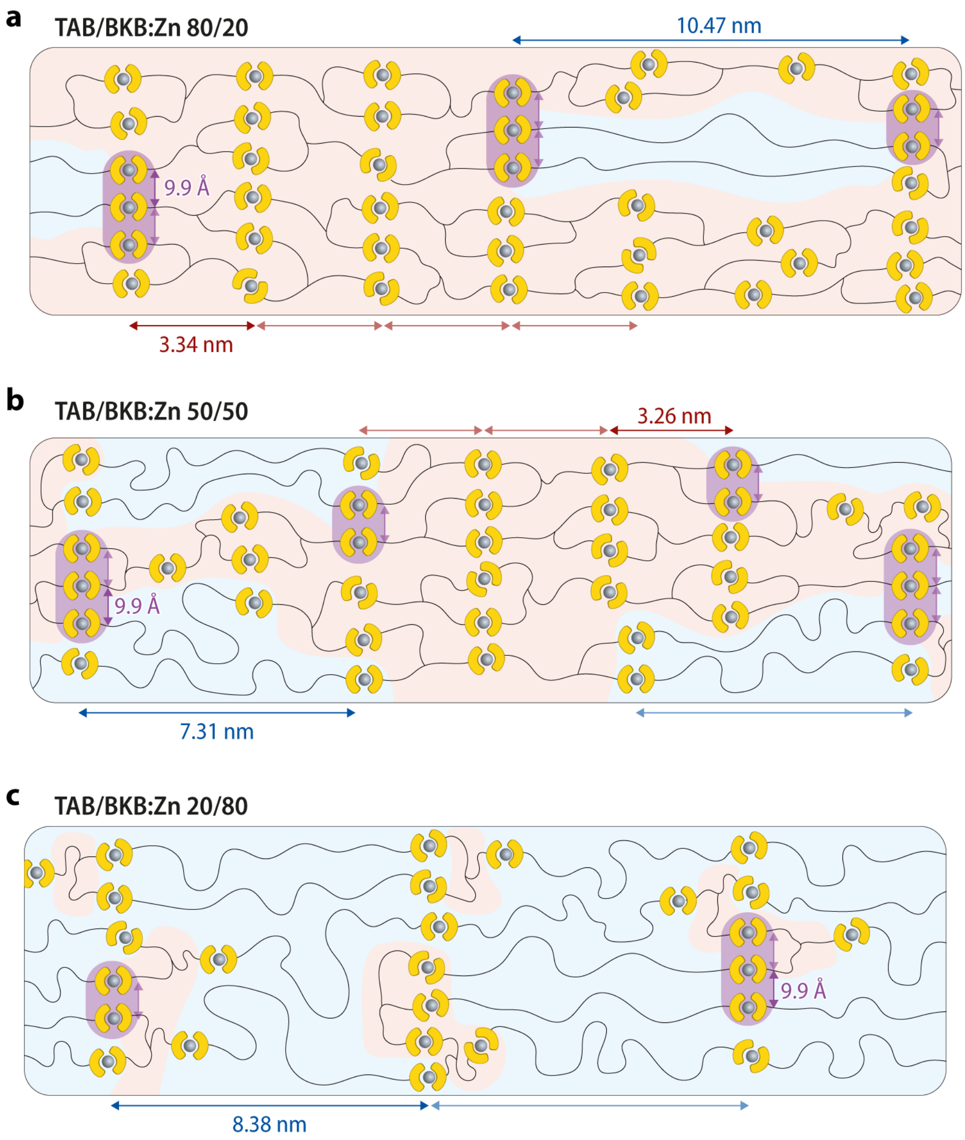

Supplementary Figure 18. Schematic representation of the possible morphologies of TAB/BKB:Zn copolymers containing TAB and BKB in a weight ratio of **(a)** 80:20, **(b)** 50:50, and **(c)** 20:80 wt/wt%. The indicated characteristic spacings were determined by analysis of the small- and wide-angle X-ray scattering (SAXS/WAXS) profiles (see Supplementary Figures 15-16 and Supplementary Table 2). The analysis of the WAXS profiles suggests that the co-assembly of the two building blocks in the presence of stoichiometric amounts of $Zn(NTf_2)_2$ leads to the formation of a mixed crystalline phase involving ligands of both monomers (purple).

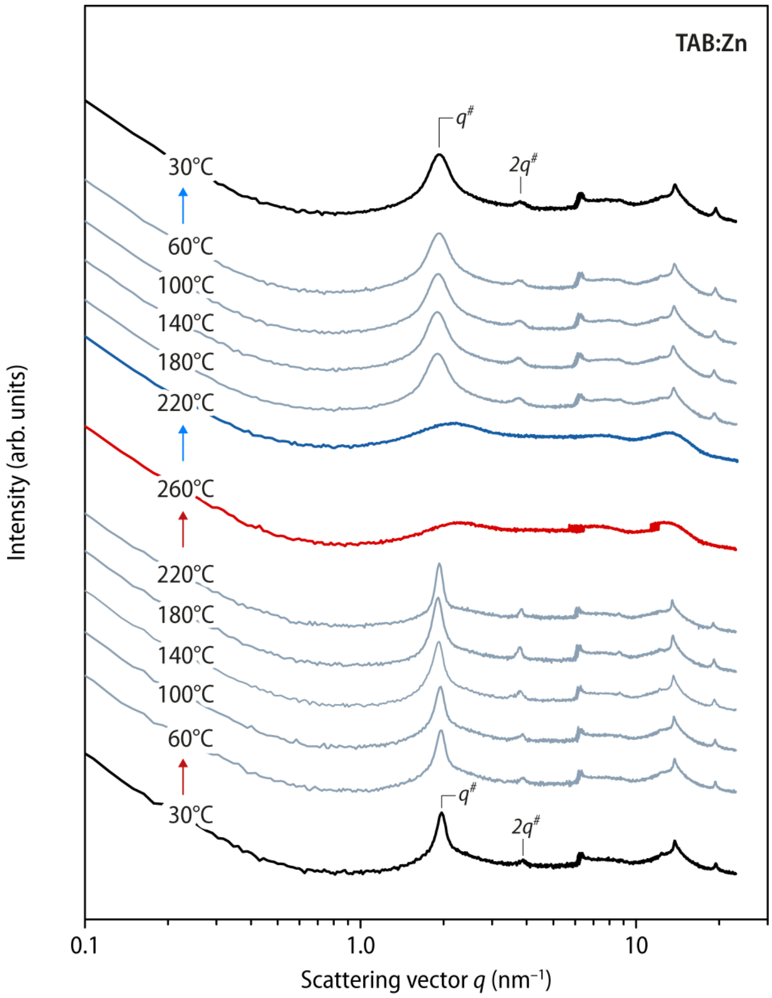

Supplementary Figure 19. Comparison of the small- and wide-angle X-ray scattering (SAXS/WAXS) profiles of a TAB:Zn film upon heating to different temperatures between 30 °C and 260 °C, as well as the scattering profiles recorded at different temperature between 220 °C and 30 °C during subsequent cooling. The heating and cooling rate for these temperature-dependent scattering experiments was 10 °C min^{-1} and samples were equilibrated at each temperature for 10 min before the data collection was started. Scattering profiles are shifted vertically for clarity.

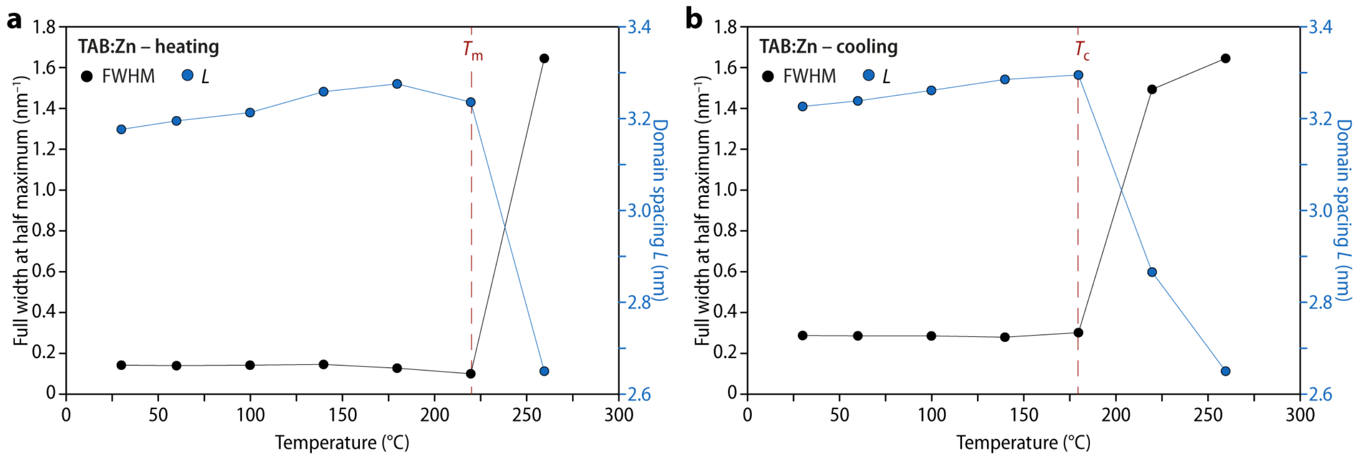

Supplementary Figure 20. Plots showing the evolution of the full width at half maximum (FWHM) of the first order Bragg diffraction peak (q^*) as determined by a least-squares fitting using a Lorentzian peak function as well as the corresponding domain spacing L ($2\pi/q^*$) as a function of the measurement temperature for the scattering experiment with TAB:Zn samples shown in Supplementary Figure 19 **(a)** upon heating, and **(b)** upon cooling.

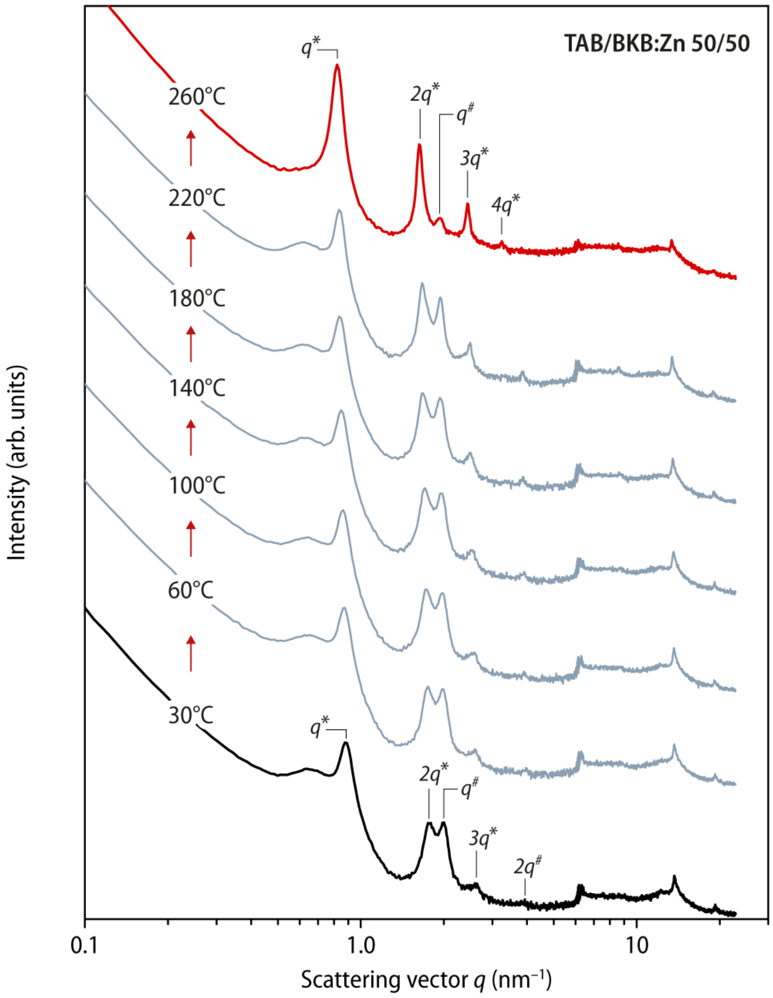

Supplementary Figure 21. Comparison of the small- and wide-angle X-ray scattering (SAXS/WAXS) profiles of the TAB/BKB:Zn copolymer containing TAB and BKB in a 50:50 wt/wt% ratio upon heating to different temperatures between 30 °C and 260 °C. The heating rate for these temperature-dependent scattering experiments was 10 °C min^{-1} and samples were equilibrated at each temperature for 10 min before the data collection was started. Scattering profiles are shifted vertically for clarity.

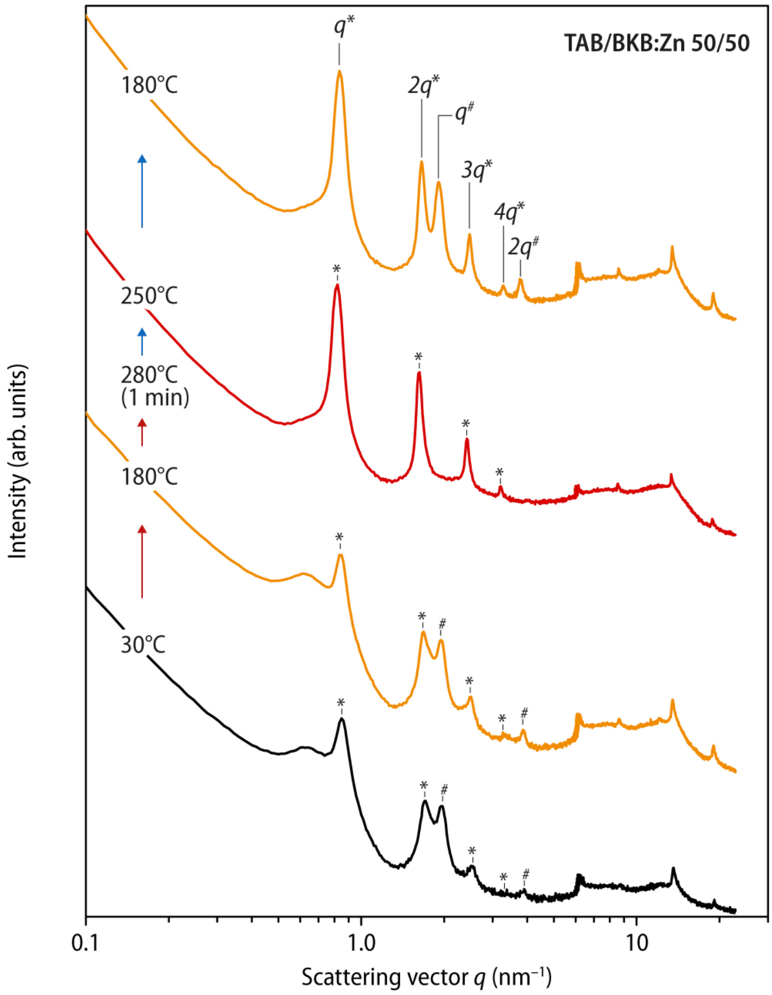

Supplementary Figure 22. Temperature-dependent SAXS/WAXS profiles for samples of the TAB/BKB:Zn copolymer containing TAB and BKB in a 50:50 wt/wt% ratio recorded at 30 and 180 °C, after cooling from the melt (1 min at 280 °C) to 250 °C, and after cooling back to 180 °C. The scattering profiles are vertically shifted for clarity and heating as well cooling rates of 10 °C min^{-1} were employed.

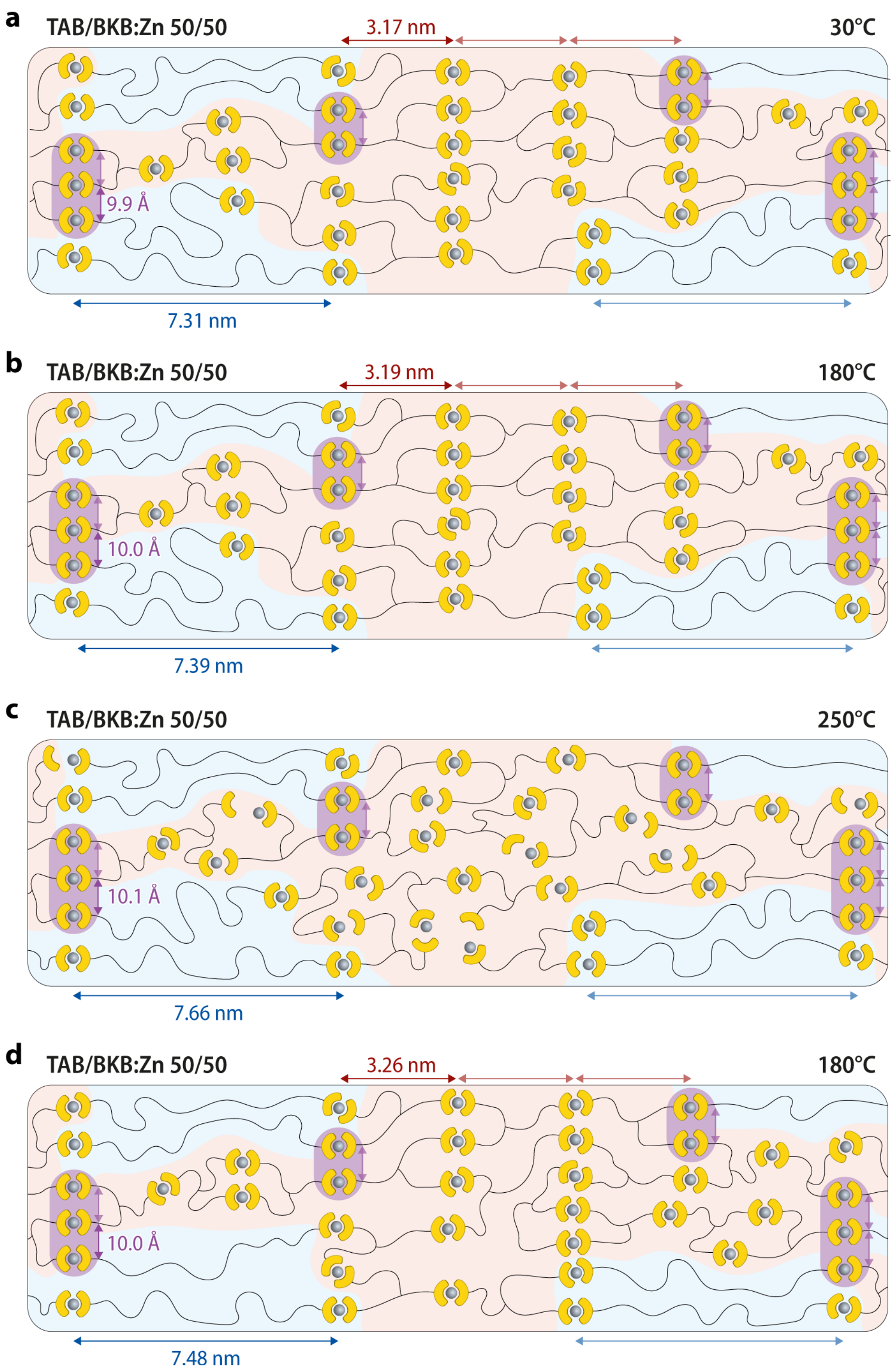

Supplementary Figure 23. Schematic representation of possible morphologies of the TAB/BKB:Zn copolymer containing TAB and BKB in a 50:50 wt/wt% ratio at temperatures of **(a)** 30 °C, **(b)** 180 °C, **(c)** after cooling to 250 °C from the melt (1 min at 280 °C), and **(d)** after cooling back to 180 °C. The indicated characteristic spacings were determined by analysis of the small- and wide-angle X-ray scattering (SAXS/WAXS) profiles (see Supplementary Figure 22 and Supplementary Table 4). The scattering data indicate that the mixed crystalline phase (purple) involving ligands of both monomers is maintained above the T_m of the TAB-rich domains, effectively hindering macrophase separation at elevated temperatures.

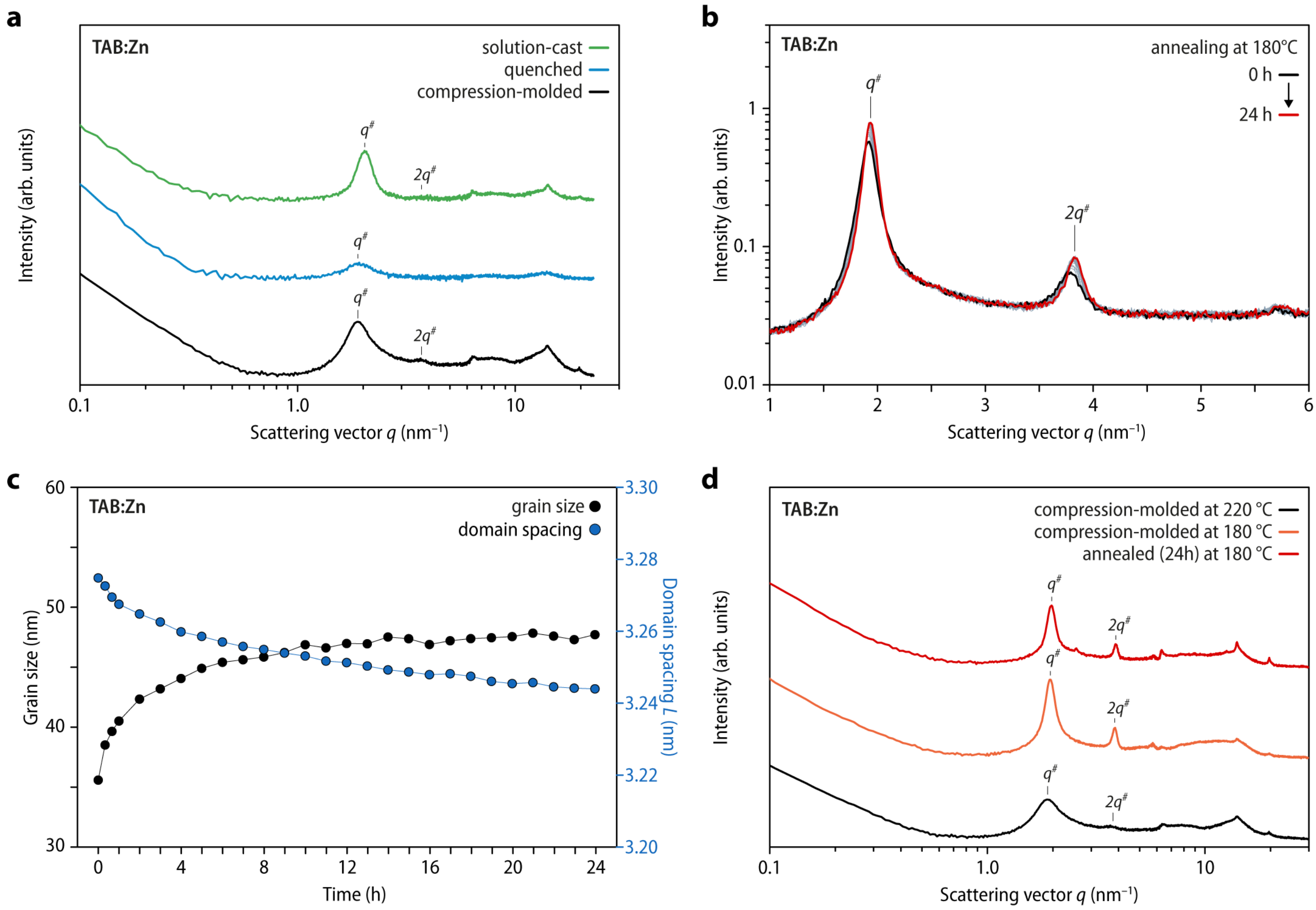

Supplementary Figure 24. (a) Comparison of the SAXS scattering profiles of TAB:Zn films obtained by solution-casting from acetonitrile and subsequent drying for 24 h in an oven at 50 °C (green), by compression molding at 220 °C (8 tons, 10 s) and quenching to room temperature (blue), and by compression molding at 220 °C (8 tons, 10 s) and cooling to room temperature at ca. 5 °C min^{-1} (black). Scattering profiles are vertically shifted for clarity. **b)** Plot showing SAXS scattering profiles of a compression-molded TAB:Zn sample (220 °C, 8 tons, 10 s) recorded during annealing at 180 °C over the course of 24 h. **c)** Evolution of the structural order in a sample of TAB:Zn as a function of annealing time at 180 °C as indicated by the grain size (black circles) and lamellar periodicity (blue circles) determined from the scattering profiles in **(b)**. The grain size (35.5 to 47.7 nm) significantly increases over time based on a Scherrer-analysis of the full width at half maximum of the first order peak (q^*), while the lamellar periodicity barely changes (3.27 to 3.24 nm). **d)** Comparison of the SAXS scattering profiles of solution-cast TAB:Zn films that were compression-molded at 220 °C (black), compression-molded at 180 °C (orange), and compression-molded at 180 °C as well as annealed in an oven at 180 °C for 24 h. Scattering profiles are vertically shifted for clarity.

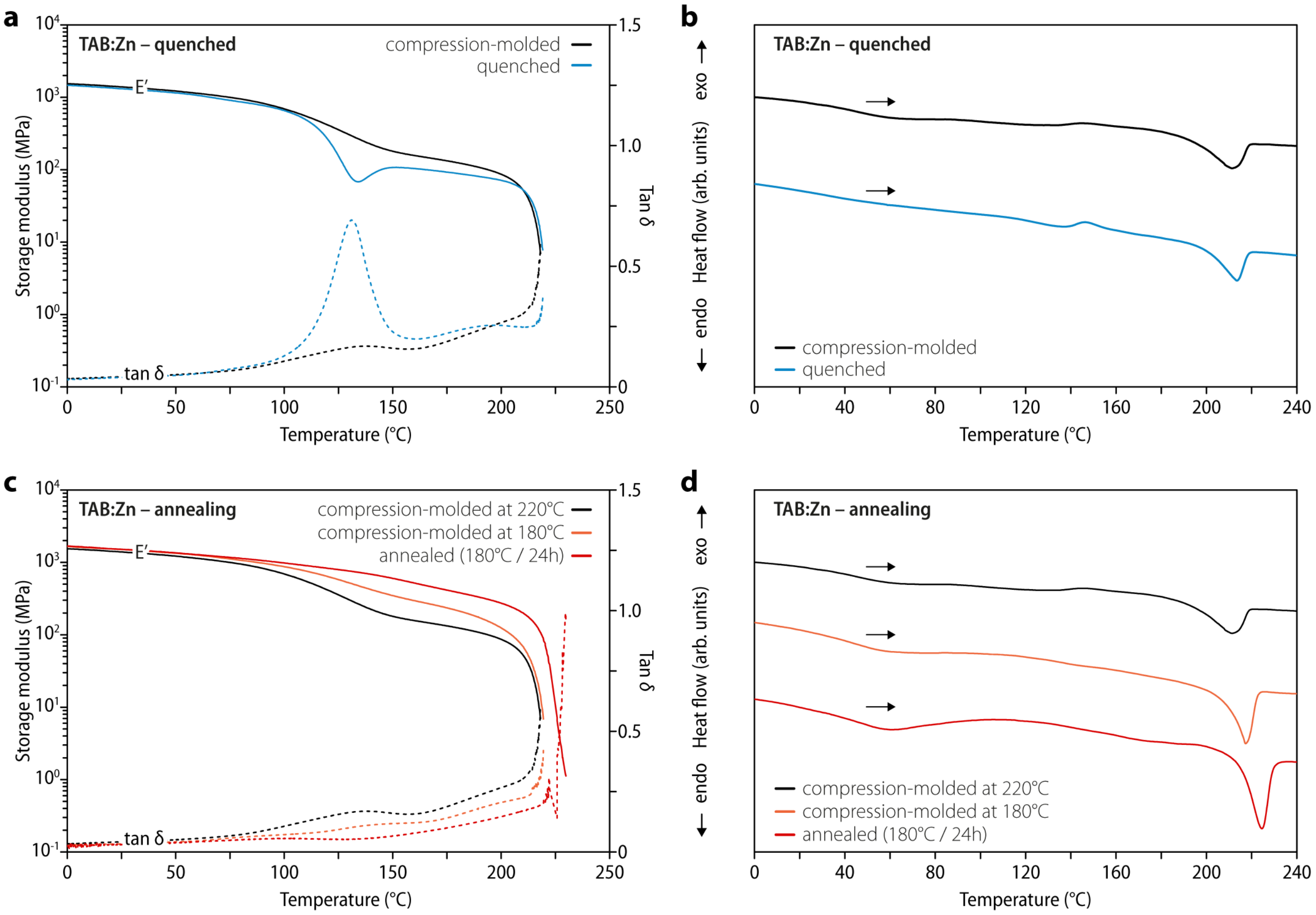

Supplementary Figure 25. (a,b) Comparison of the **(a)** dynamic mechanical analysis (DMA) and **(b)** differential scanning calorimetry (DSC) traces of compression-molded samples of TAB:Zn (black) and the traces of samples that were thermally quenched (blue). **(c,d)** Comparison of the **(c)** DMA and **(b)** DSC traces of solution-cast TAB:Zn films that were compression-molded at 220 °C (black), compression-molded at 180 °C (orange), and compression-molded at 180 °C as well as annealed in an oven at 180 °C for 24 h (red). Shown in **(a)** and **(c)** are the storage moduli (solid line) and the tan δ (dashed line) traces. A heating rate of 10 °C min^{-1} was used for the DSC experiments shown in **(b)** and **(d)** and the traces are vertically shifted for clarity.

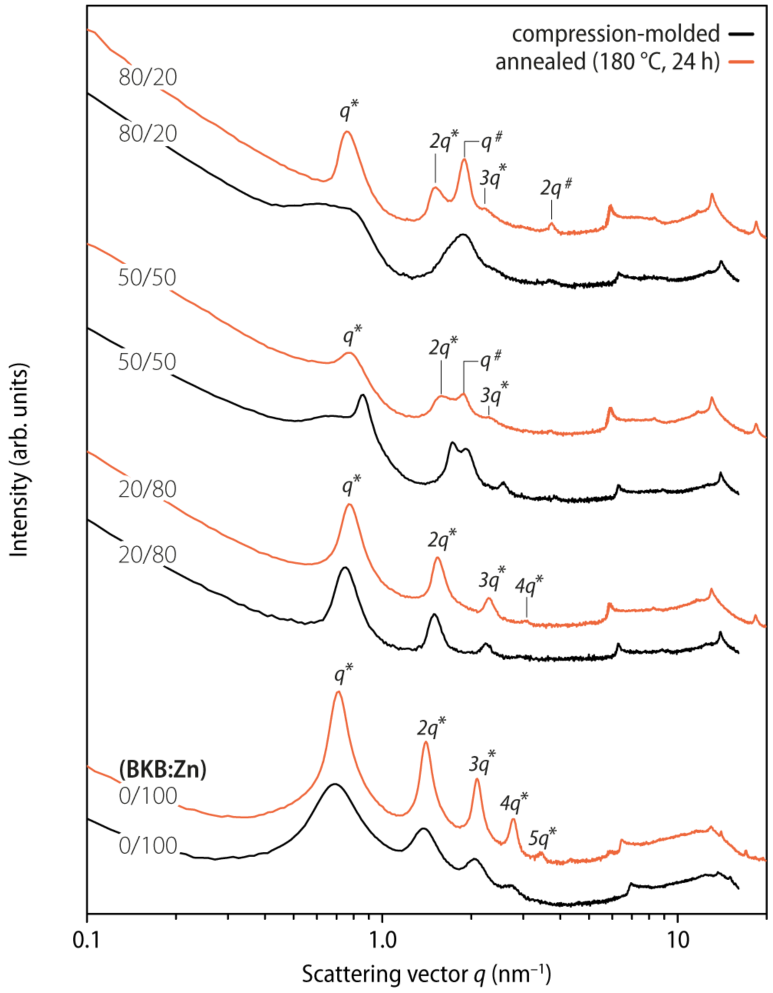

Supplementary Figure 26. Small- and wide-angle X-ray scattering (SAXS/WAXS) profiles of the neat metallo-supramolecular polymer BKB:Zn and the TAB/BKB:Zn copolymers with the indicated TAB/BKB weight ratio (wt/wt%) recorded with samples that were compression-molded (black lines) and samples that were additionally annealed for 24 h in an oven at a temperature of 180 °C. Scattering profiles are shifted vertically for clarity.

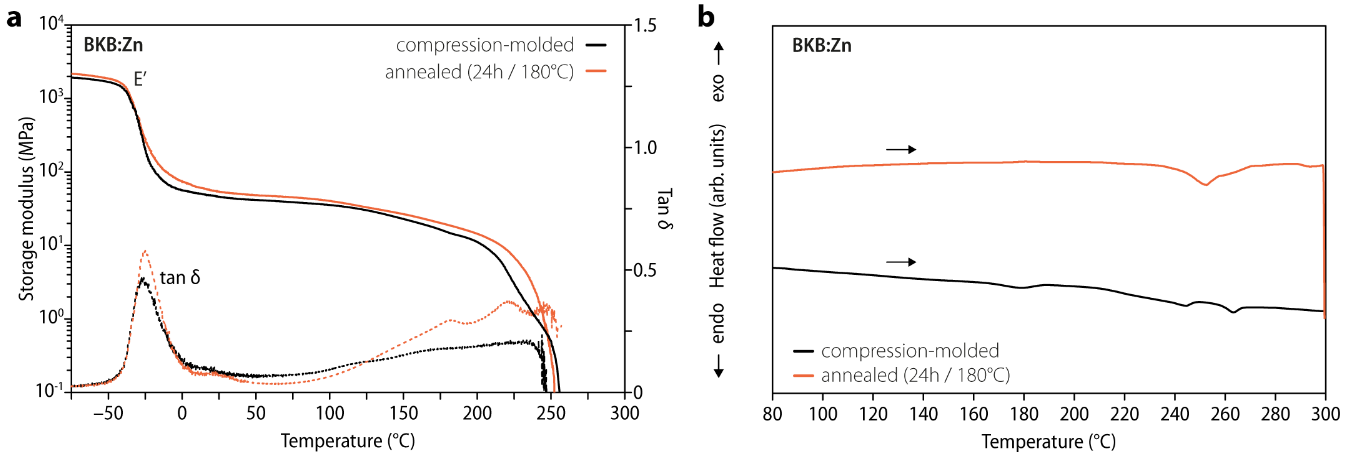

Supplementary Figure 27. Comparison of the **(a)** dynamic mechanical analysis (DMA) and **(b)** differential scanning calorimetry (DSC) traces of compression-molded samples of BKB:Zn (black) and the traces of samples that were annealed in an oven at 180 °C for 24 h. The melting transition of annealed BKB:Zn samples appears comparable to the transition observed for solvent-cast samples (Supplementary Figure 6). Shown in **(a)** are the storage moduli (solid line) and the tan δ (dashed line) traces. A heating rate of 10 °C min^{-1} was used for the DSC experiments shown in **(b)** and the traces are vertically shifted for clarity.

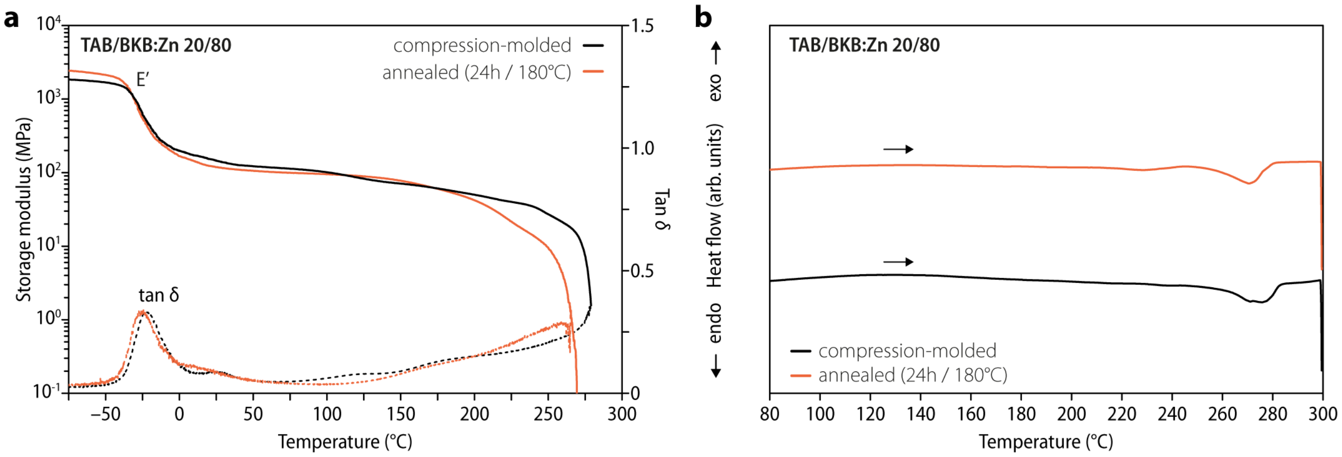

Supplementary Figure 28. Comparison of the **(a)** dynamic mechanical analysis (DMA) and **(b)** differential scanning calorimetry (DSC) traces of compression-molded samples of TAB/BKB:Zn copolymer containing TAB and BKB in a 20:80 wt/wt% ratio (black) and the traces of samples that were annealed in an oven at 180 °C for 24 h. Shown in **(a)** are the storage moduli (solid line) and the tan δ (dashed line) traces. A heating rate of 10 °C min^{-1} was used for the DSC experiments shown in **(b)** and the traces are vertically shifted for clarity.

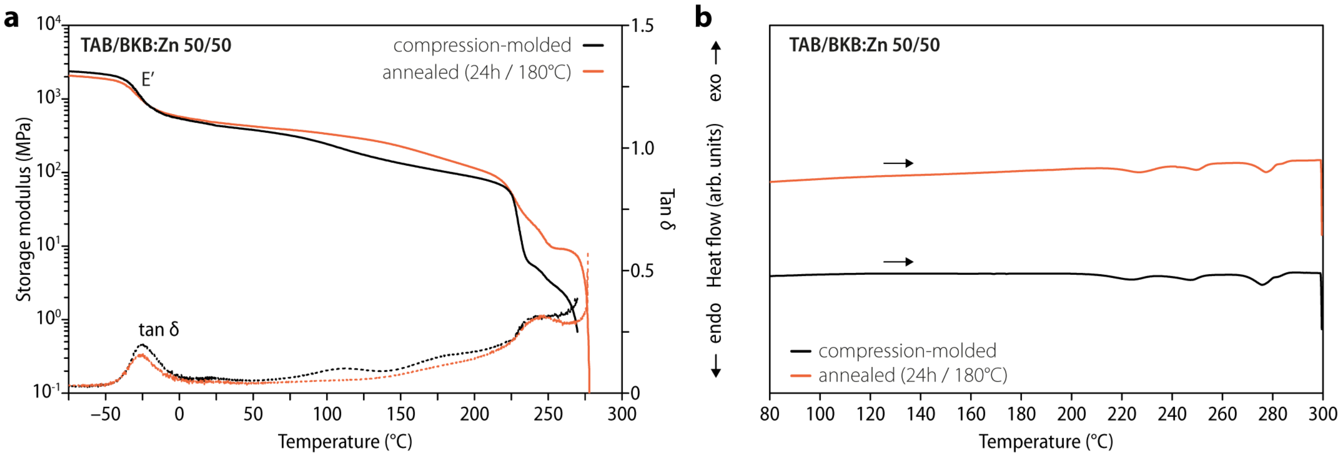

Supplementary Figure 29. Comparison of the **(a)** dynamic mechanical analysis (DMA) and **(b)** differential scanning calorimetry (DSC) traces of compression-molded samples of TAB/BKB:Zn copolymer containing TAB and BKB in a 50:50 wt/wt% ratio (black) and the traces of samples that were annealed in an oven at 180 °C for 24 h. Shown in **(a)** are the storage moduli (solid line) and the tan δ (dashed line) traces. A heating rate of 10 °C min^{-1} was used for the DSC experiments shown in **(b)** and the traces are vertically shifted for clarity.

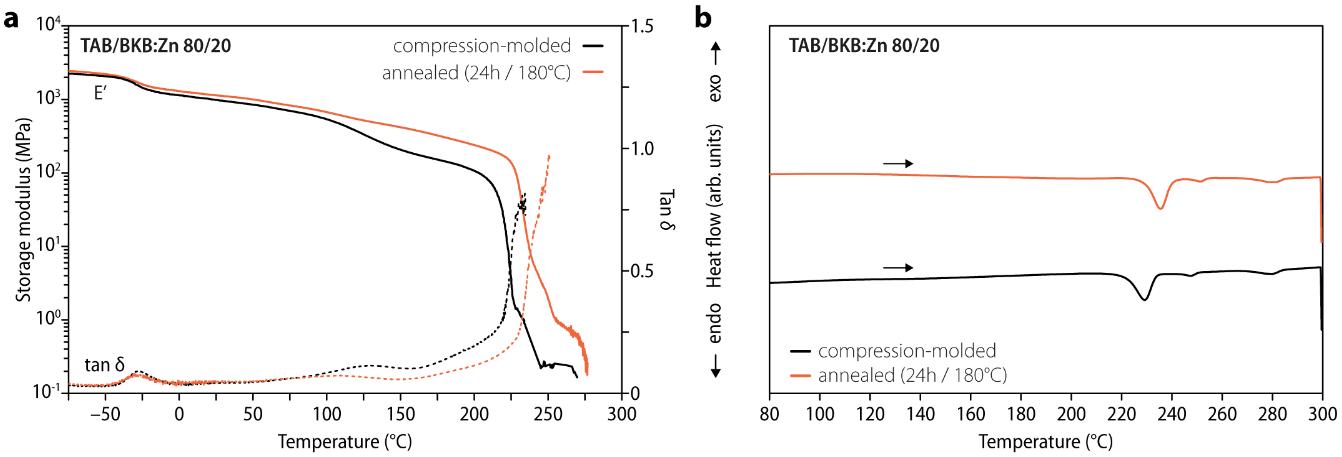

Supplementary Figure 30. Comparison of the **(a)** dynamic mechanical analysis (DMA) and **(b)** differential scanning calorimetry (DSC) traces of compression-molded samples of TAB/BKB:Zn copolymer containing TAB and BKB in a 80:20 wt/wt% ratio (black) and the traces of samples that were annealed in an oven at 180 °C for 24 h. Shown in **(a)** are the storage moduli (solid line) and the tan δ (dashed line) traces. A heating rate of 10 °C min^{-1} was used for the DSC experiments shown in **(b)** and the traces are vertically shifted for clarity.

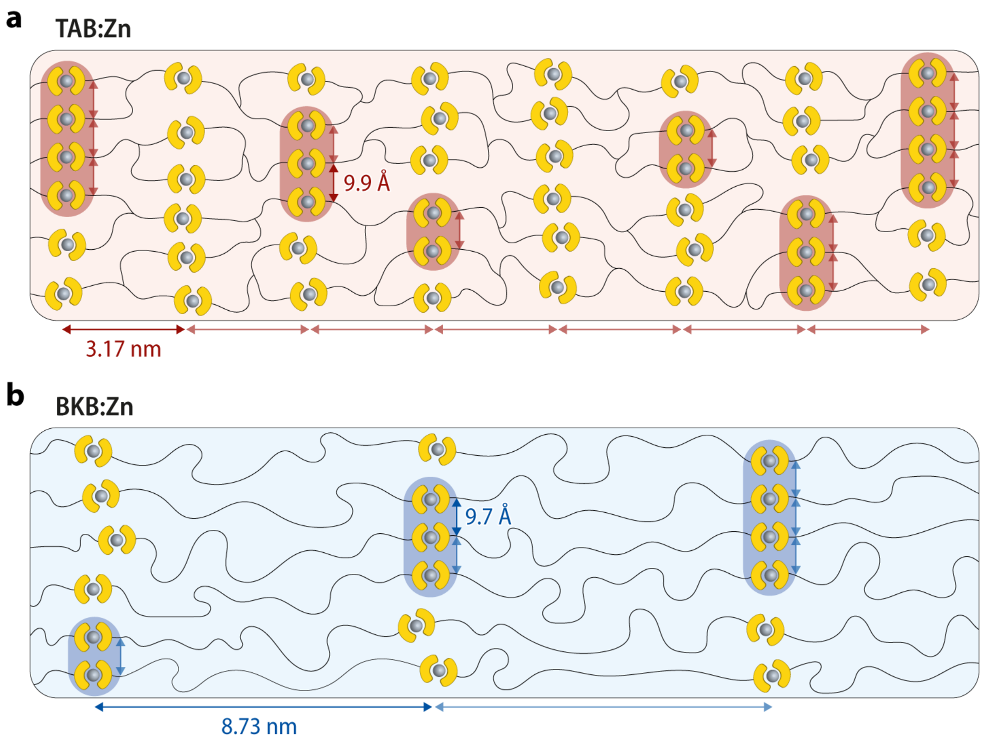

Supplementary Figure 31. Schematic representation of the possible morphologies of annealed (180 °C, 24 h) samples of the neat metallosupramolecular polymers **(a)** TAB:Zn and **(b)** BKB:Zn. The indicated characteristic spacings were determined by analysis of the small- and wide-angle X-ray scattering (SAXS/WAXS) profiles (see Supplementary Figures 24, 26, and Supplementary Table 9). The WAXS profiles show characteristic spacings that indicate the presence of crystalline domains of the metal ligand complexes in the neat metallosupramolecular polymers, which are highlighted in dark red and blue, respectively.

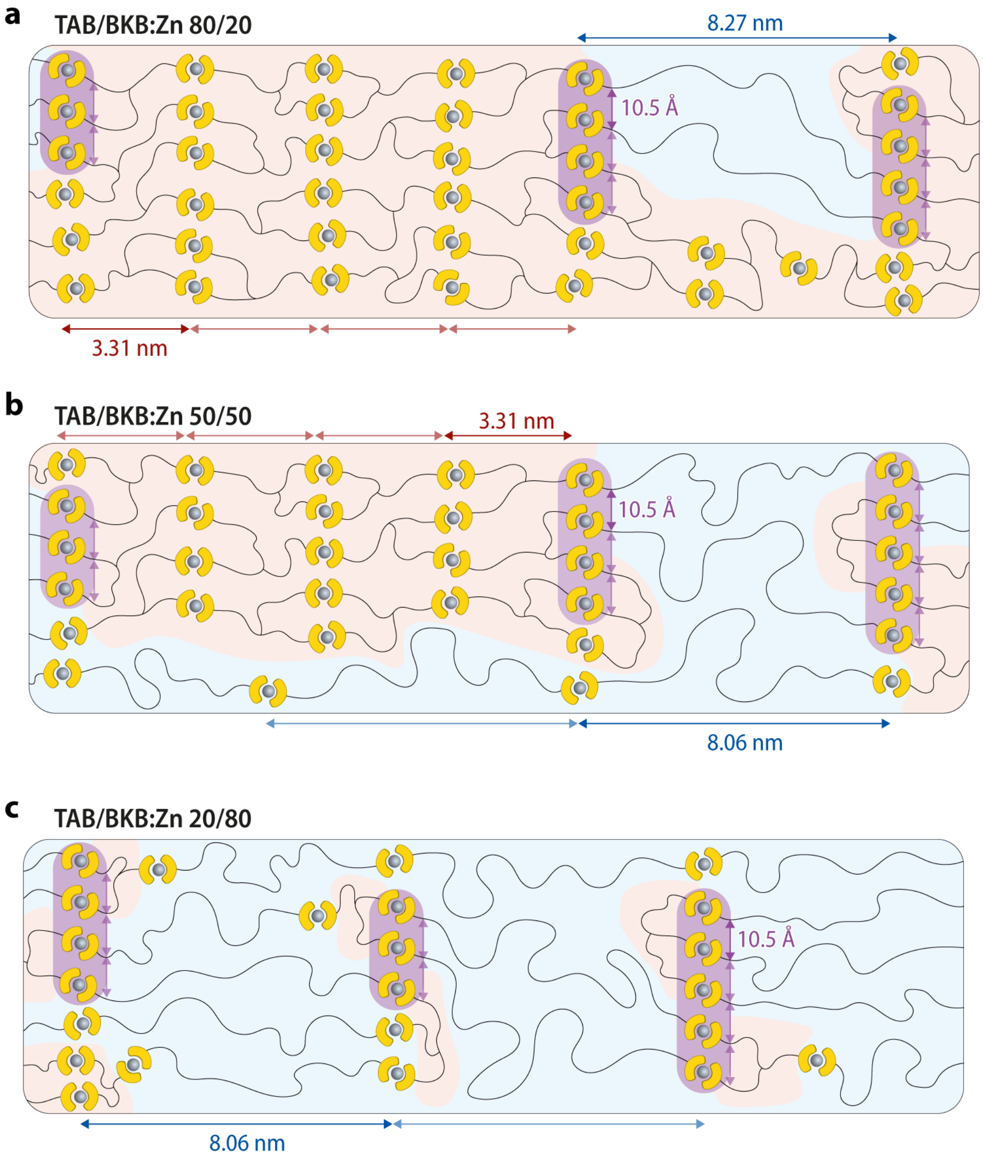

Supplementary Figure 32. Schematic representation of the possible morphologies of annealed (180 °C, 24 h) samples of TAB/BKB:Zn copolymers containing TAB and BKB in a weight ratio of **(a)** 80:20, **(b)** 50:50, and **(c)** 20:80 wt/wt%. The indicated characteristic spacings were determined by analysis of the small- and wide-angle X-ray scattering (SAXS/WAXS) profiles (see Supplementary Figures 24, 26, and Supplementary Table 9).

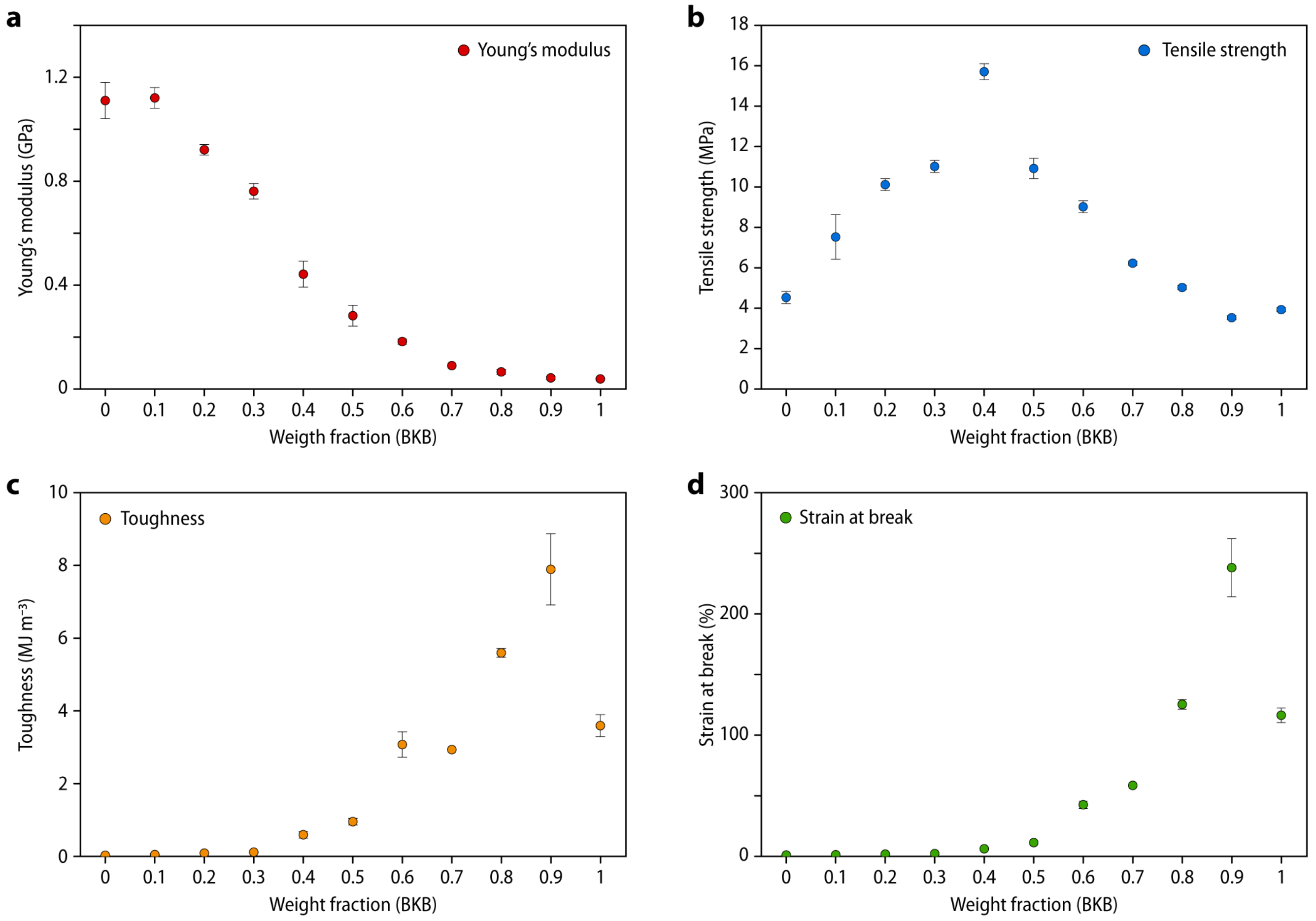

Supplementary Figure 33. Plots of the **(a)** Young's modulus, **(b)** tensile strength, **(c)** toughness, and **(d)** strain at break of samples of the metallosupramolecular polymers TAB:Zn, BKB:Zn, and their copolymers TAB/BKB:Zn as a function of the weight fraction of BKB. The mechanical properties were determined by uniaxial stress-strain experiments at 25 °C with a strain rate of 1% min^{-1}. The data represent averages of 3–7 individual measurements with standard deviation.

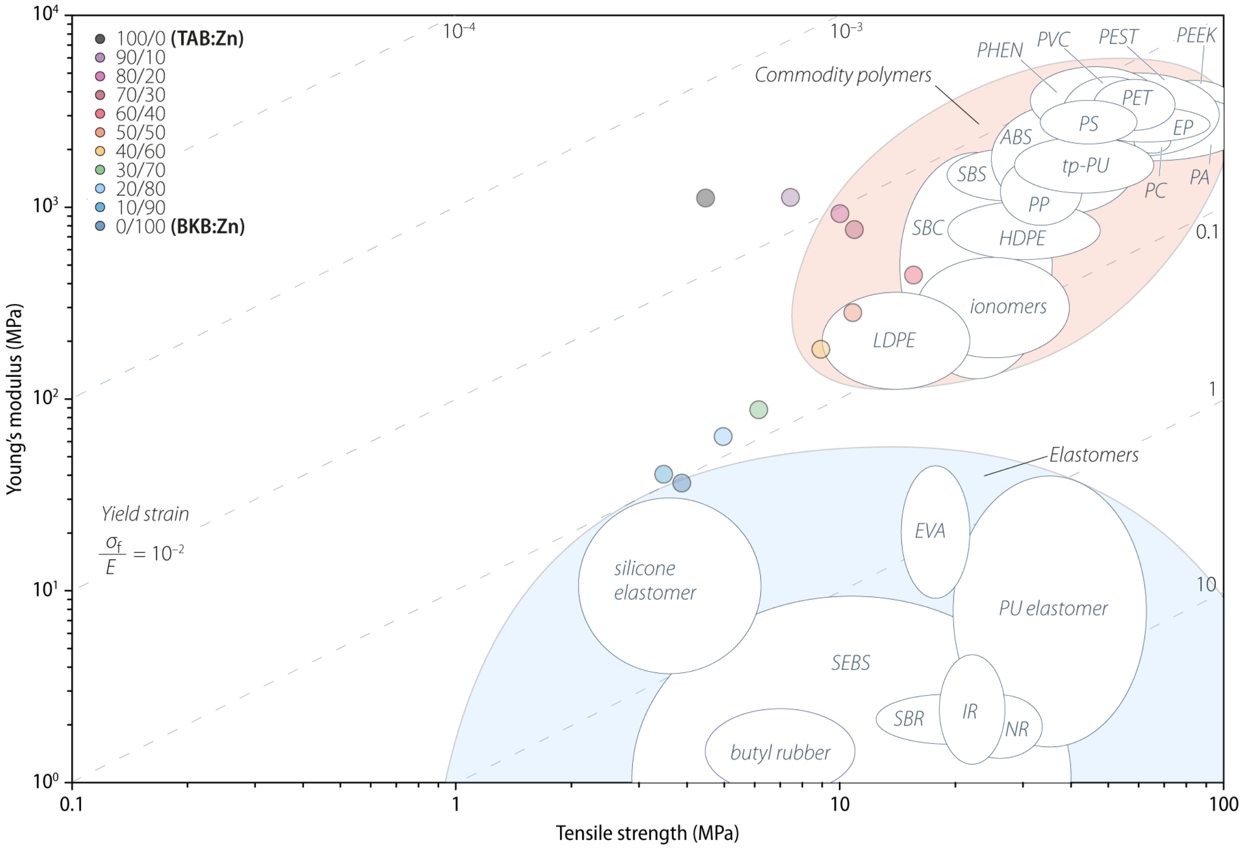

Supplementary Figure 34. Ashby (materials selection) plot showing the Young's moduli and tensile strengths of the MSPs and copolymers reported herein as well as the properties attainable with typical examples of elastomers and commodity polymers.[1,2] The following abbreviations are used: poly(acrylonitrile-*co*-butadiene-*co*-styrene) (ABS), epoxies (EP), ethylene-vinyl acetate copolymer (EVA), high density polyethylene (HDPE), isoprene rubber (IR), low-density polyethylene (LDPE), natural rubber (NR), polyamides (PA), polycarbonate (PC), polyethylene terephthalate (PET), polyesters (PEST), polyetheretherketone (PEEK), phenolics (PHEN), polypropylene (PP), polystyrene (PS), polyurethane elastomers (PU elastomer), polyvinylchloride (PVC), styrene-ethylene-butylene-styrene triblock copolymer (SEBS), styrene-butadiene block copolymer (SBC), poly(styrene-*co*-butadiene) (SBR), styrene-butadiene-styrene triblock copolymer (SBS), and polyurethane thermoplastics (tp-PU).

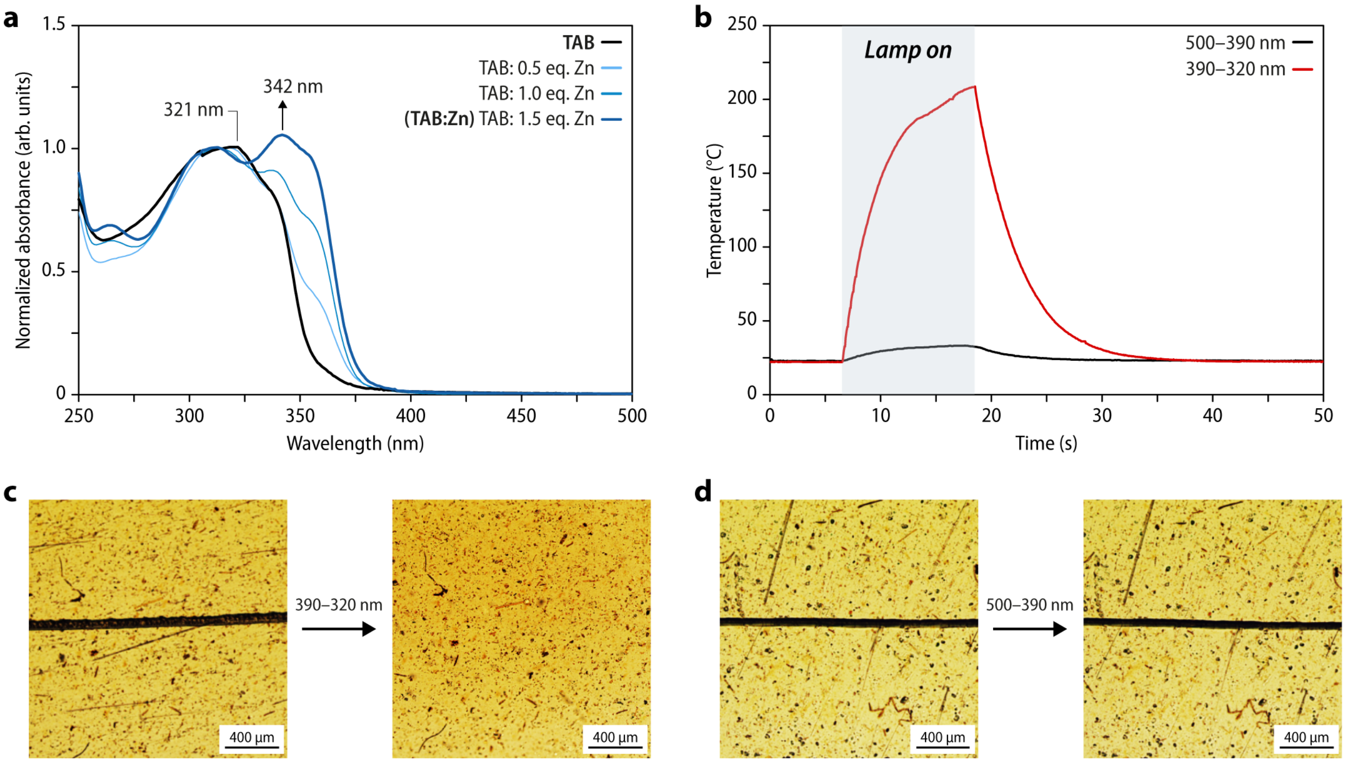

Supplementary Figure 35. (a) Comparison of solid state UV-vis absorption spectra recorded with films (< 10 µm) spin coated on quartz slides of TAB and of TAB:Zn with different amounts of the $Zn(NTf_2)_2$ metal salt. **(b)** Surface temperature measured using an infrared camera upon irradiating the TAB:Zn film with light of a wavelength of 320-390 nm (red) or 390-500 nm (black) for the duration of 12 s with an intensity of 320 mW cm^{-2}. **(c)** Microscopy images showing the optical healing of a cut in a film of TAB:Zn. The 100-µm-thin film was cut to a depth of ca. 30% of the total film thickness (left) and subsequently exposed to ultraviolet radiation (320–390 nm, 320 mW cm^{-2}, 12 s) (right). **(d)** Microscopy images of a 100-µm-thin TAB:Zn film with a well-defined cut to a depth of ca. 30% of the total film thickness before (left) and after (right) irradiation with light in the wavelength range of 390–500 nm (320 mW cm^{-2}, 12 s).

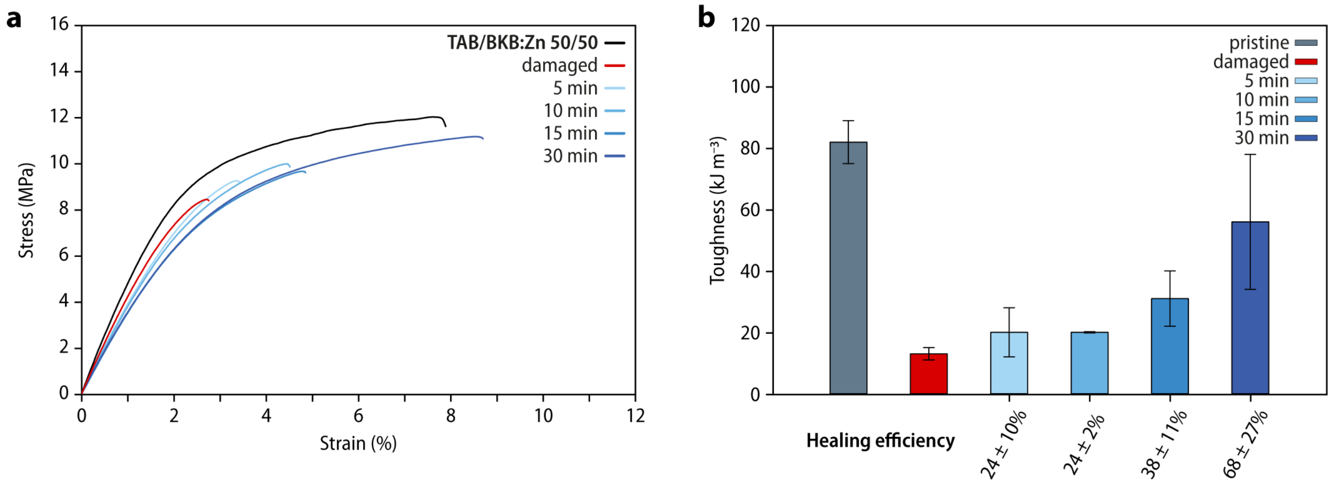

Supplementary Figure 36. **(a)** Comparison of representative stress-strain curves of samples of TAB/BKB:Zn copolymer films with a TAB:BKB ratio of 50:50 wt/wt% in the pristine state (black), after applying a cut to a depth of ca. 30% of the total film thickness (red), and after healing by heating to 220 °C for 5, 10, 15, and 30 min (blue). The stress-strain experiments were carried out at 25 °C with a strain rate of 1% min^{-1}. **(b)** Comparison of the toughness of samples of the TAB/BKB:Zn copolymer films with TAB:BKB ratios of 50:50 wt/wt% in the pristine state, after damaging sample by cutting to a depth of ca. 40% of the total film thickness, and after thermal healing by locally heating to 220 °C for 5, 10, 15, and 30 min (error bars represent the standard deviation of n = 3–4 individual measurements).

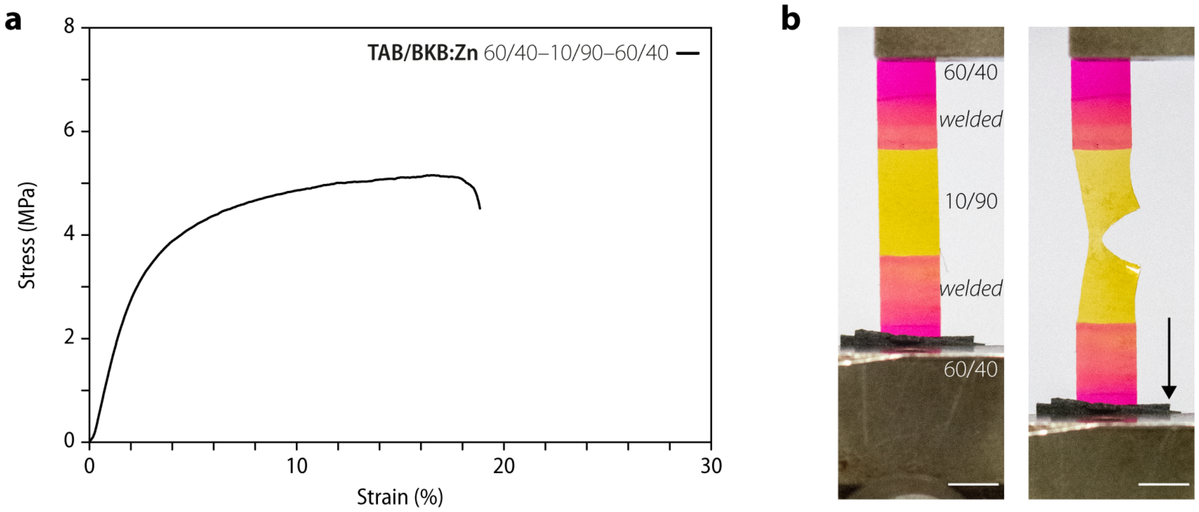

Supplementary Figure 37. (a) Representative stress-strain curve of a segmented sample obtained by welding copolymer films with TAB:BKB ratios of 60:40 wt/wt% with a film with a TAB:BKB ratio of 10:90 wt/wt%. The stress-strain experiments were carried out at 25 °C with a strain rate of 1% min^{-1}. **(b)** Photograph of the welded copolymer sample with TAB:BKB ratios of 60:40 wt/wt% (stiff, red-dyed) and 10:90 wt/wt% (soft, yellow-dyed). The modulus is lowest in the segment with a TAB:BKB ratio of 10:90 wt/wt% and samples fail there upon uniaxial tensile deformation.

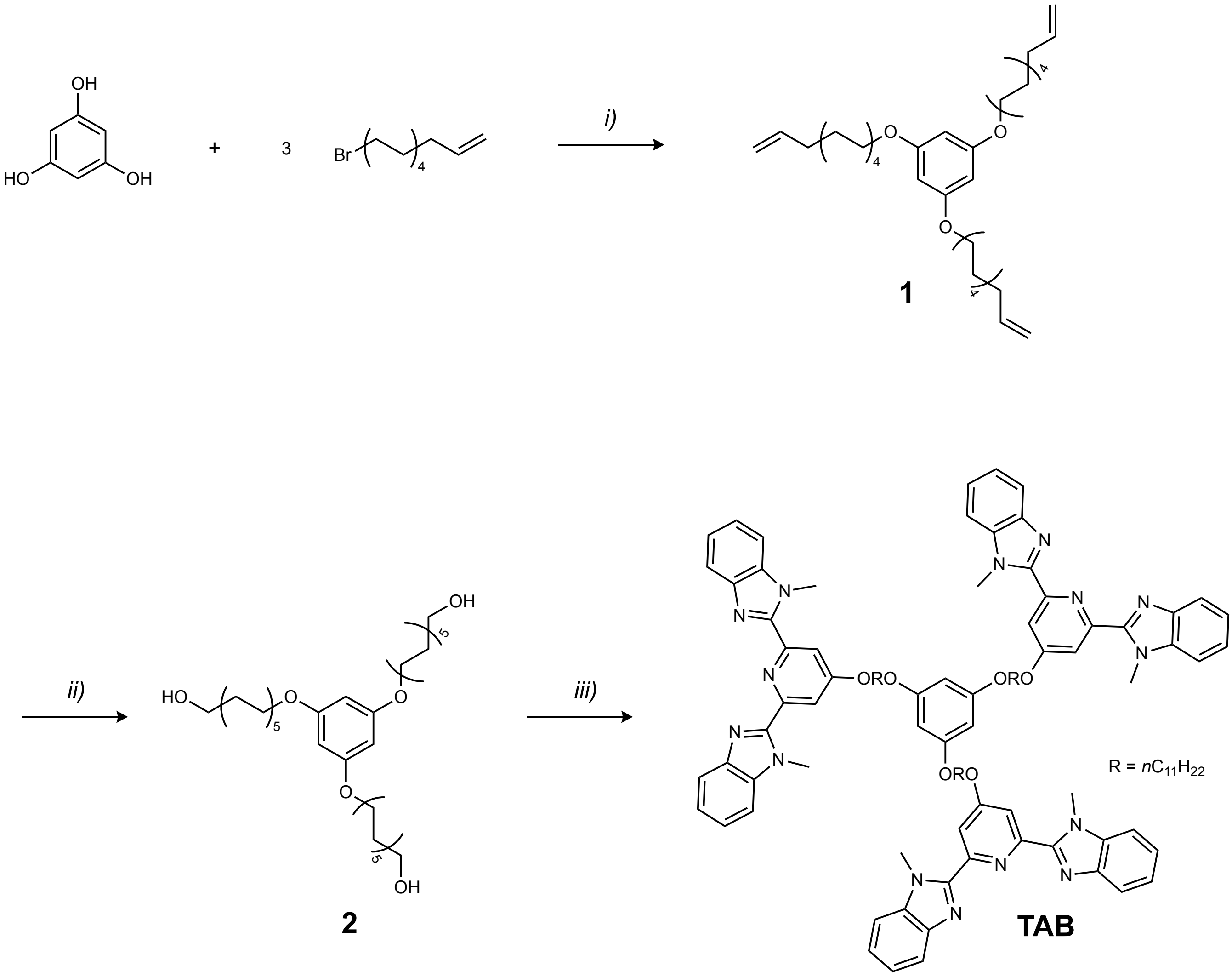

Supplementary Figure 38. Synthetic route toward the new low-molecular-weight trifunctional building block (TAB) with 2,6-bis(1′-methylbenzimidazolyl)pyridine ligands. *Reagents and conditions: i)* K_2CO_3, DMF, 40%; *ii)* 9-BBN, THF; *then* H_2O_2, NaOH, water, 87%; *iii)* 4-hydroxy-2,6-bis(*N*-methylbenzimidazol-2'-yl)pyridine, PPh_3, DEAD, THF/toluene, 61%.

2. Supplementary Tables 1−11

Supplementary Table 1. Thermomechanical properties of the neat metallosupramolecular polymers TAB:Zn and BKB:Zn, as well as of the TAB/BKB:Zn copolymers with the indicated TAB/BKB weight ratio (wt/wt%).

Composition	Storage Modulus (MPa)				Failure Temperature
	At –80 °C	At 25 °C	At 170 °C	At 250 °C	(°C)
**TAB:Zn**	n.a.	1419 ± 66	140 ± 3	n.a.	215–220
**TAB/BKB:Zn (90/10)**	2296 ± 205	1318 ± 129	188 ± 22	n.a.	225–235
**TAB/BKB:Zn (80/20)**	2220 ± 124	974 ± 29	157 ± 3	< 0.50*	270–275
**TAB/BKB:Zn (70/30)**	2202 ± 125	908 ± 16	161 ± 4	< 0.50*	270–275
**TAB/BKB:Zn (60/40)**	2248 ± 103	701 ± 21	149 ± 5	< 0.50*	270–275
**TAB/BKB:Zn (50/50)**	2443 ± 98	447 ± 22	114 ± 4	3.3 ± 0.2	275–280
**TAB/BKB:Zn (40/60)**	2206 ± 252	238 ± 13	80 ± 6	13 ± 2	280–290
**TAB/BKB:Zn (30/70)**	2088 ± 151	176 ± 8	77 ± 3	31 ± 4	280–285
**TAB/BKB:Zn (20/80)**	1996 ± 136	145 ± 1	62 ± 1	25 ± 2	280–285
**TAB/BKB:Zn (10/90)**	1727 ± 3	54 ± 4	33 ± 2	17 ± 1	275–280
**BKB:Zn**	1731 ± 208	45 ± 2	17 ± 1	0.55 ± 0.04	260–265

Data represent averages of n = 3–5 individual DMA measurements ± standard deviation. *Static force is out of the measuring range.

Supplementary Table 2. Data of SAXS and WAXS analysis for neat metallosupramolecular polymers TAB:Zn and BKB:Zn, as well as of the TAB/BKB:Zn copolymers with the indicated TAB/BKB weight ratio (wt/wt%).

**Composition**	q^* (nm^{-1})	L^* (nm)	$q^\#$ (nm^{-1})	$L^\#$ (nm)	q^0_{WAXS} (nm^{-1})	L^0 (Å)
**TAB:Zn**	–	–	1.91	3.29	6.39	9.8
**TAB/BKB:Zn (90/10)**	0.85	7.39	1.94	3.24	6.35	9.9
**TAB/BKB:Zn (80/20)**	0.60	10.4	1.88	3.34	6.35	9.9
**TAB/BKB:Zn (70/30)**	0.85	7.39	1.96	3.21	6.35	9.9
**TAB/BKB:Zn (60/40)**	0.85	7.39	1.94	3.24	6.35	9.9
**TAB/BKB:Zn (50/50)**	0.86	7.31	1.93	3.26	6.35	9.9
**TAB/BKB:Zn (40/60)**	0.78	8.06	1.93	3.26	6.35	9.9
**TAB/BKB:Zn (30/70)**	0.80	7.85	1.93	3.26	6.35	9.9
**TAB/BKB:Zn (20/80)**	0.75	8.37	–	–	6.35	9.9
**TAB/BKB:Zn (10/90)**	0.77	8.16	–	–	6.35	9.9
**BKB:Zn**	0.70	8.98	–	–	7.02	8.9

q^* and $q^\#$ are the scattering vectors of the primary Bragg diffraction peak of each pattern and the values obtained at the maximum intensity of the primary Bragg diffraction peak to each Bragg diffraction maximum is given. q^0_{WAXS} is the scattering vector of the primary WAXS diffraction peak of each pattern and the values obtained at the maximum intensity of the primary peak are given. The period L^*, $L^\#$, and L^0 are calculated as $2\pi/q$ using q^*, $q^\#$, or q^0_{WAXS}, respectively.

Supplementary Table 3. Data of the analysis of temperature-dependent SAXS measurements of the TAB:Zn films.

**Temperature** (°C)	$q^{\#}$ (nm^{-1})	***L*** (nm)	**FWHM** (nm^{-1})	**Grain Size** (nm)
30 (Heating)	1.98	3.18	0.139	40.7
60 (Heating)	1.97	3.19	0.136	41.6
100 (Heating)	1.96	3.21	0.139	40.7
140 (Heating)	1.93	3.26	0.142	39.8
180 (Heating)	1.92	3.28	0.124	45.6
220 (Heating)	1.94	3.24	0.096	58.9
260 (Heating)	2.37	2.65	1.64	3.4
220 (Cooling)	2.19	2.86	1.49	3.8
180 (Cooling)	1.91	3.29	0.298	19.0
140 (Cooling)	1.91	3.29	0.276	20.5
100 (Cooling)	1.93	3.26	0.282	20.1
60 (Cooling)	1.94	3.24	0.282	20.1
30 (Heating)	1.95	3.23	0.284	19.9

q^* is the scattering vector of the primary Bragg diffraction peak and the value obtained at the center of a Lorentzian distribution fit to each Bragg diffraction maximum is given. The period L is calculated as $2\pi/q$ using q^*. The full width at half maximum (FWHM) is taken from the parameters of the Lorentzian distribution giving the best fit. Grain sizes are calculated based on a Scherrer-analysis of the FWHM of the first order diffraction peak with $0.9 \times (2\pi/\text{FWHM})$.

Supplementary Table 4. Data of the analysis of temperature-dependent SAXS measurements of the TAB/BKB:Zn copolymer containing TAB and BKB in a 50:50 (wt/wt%) ratio.

**Temperature**	q^*	L^*	**FWHM***	**Grain Size***	$q^\#$	$L^\#$	**FWHM**$^\#$	**Grain Size**$^\#$	q^0_{WAXS}	L^0
	(nm^{-1})	(nm)	(nm^{-1})	(nm)	(nm^{-1})	(nm)	(nm^{-1})	(nm)	(nm^{-1})	(Å)
30 °C (Heating)	0.86	7.31	0.081	69.8	1.98	3.17	0.176	32.1	6.33	9.9
180 °C (Heating)	0.85	7.39	0.078	72.5	1.97	3.19	0.176	32.1	6.31	10.0
250 °C (Cooling from 280 °C)	0.82	7.66	0.044	128.5	–	–	–	–	6.23	10.1
180 °C (Cooling from 250 °C)	0.84	7.48	0.046	123	1.93	3.26	0.117	48.3	6.28	10.0

q^* and $q^\#$ are the scattering vector of the primary Bragg diffraction peak of each pattern and the value obtained at the center of a Lorentzian distribution fit to each Bragg diffraction maximum is given. q^0_{WAXS} is the scattering vector of the primary WAXS diffraction peak of each pattern and the values obtained at the maximum intensity of the primary peak are given. The periods L^*, $L^\#$, and L^0 are calculated as $2\pi/q$ using q^*, $q^\#$, or q^0_{WAXS}, respectively. The full width at half maximum (FWHM) is taken from the parameters of the Lorentzian distribution giving the best fit. Grain sizes are calculated based on an analysis of the FWHM of the first order diffraction peak using the Scherrer equation with $0.9 \times (2\pi/\mathrm{FWHM})$.

Supplementary Table 5. Data obtained by analysis of the SAXS/WAXS scattering profiles for TAB:Zn films with a varying processing history, *i.e.*, solvent-cast films, compression molded films (220 °C, 8 tons, 10 s) that were quenched, compression molded films that were treated at 220 °C (8 tons, 10 s) or 180 °C (8 tons, 30 s) and subsequently cooled at a rate of ca. 5 °C min^{-1}, and compression molded films (180 °C, 8 tons, 30 s) that were further annealed at 180 °C in an oven for 24 h.

**TAB:Zn sample**	$q^{\#}$ (nm^{-1})	L (nm)	**FWHM** (nm^{-1})	**Grain Size** (nm)
Solvent-Cast	2.05	3.05	0.271	20.9
Compression-molded 220 °C / fast cooling	1.93	3.26	0.623	9.1
Compression-molded 220 °C / slow cooling	1.91	3.29	0.413	13.7
Compression-molded 180 °C / slow cooling	1.96	3.21	0.113	50.0
Compression-molded 180°C / 24 h	1.98	3.17	0.119	47.5

q^* is the scattering vector of the primary Bragg diffraction peak and the value obtained at the center of a Lorentzian distribution fit to each Bragg diffraction maximum is given. The period L is calculated as $2\pi/q$ using q^*. The full width at half maximum (FWHM) is taken from the parameters of the Lorentzian distribution giving the best fit. Grain sizes are calculated based on a Scherrer-analysis of the FWHM of the first order diffraction peak with $0.9 \times (2\pi/\text{FWHM})$.

Supplementary Table 6. Structural data determined by analysis of the SAXS/WAXS scattering profiles recorded at 180 °C with a TAB:Zn film after different annealing times.

**Time at 180 °C** (h)	$q^{\#}$ (nm^{-1})	*L* (nm)	**FWHM** (nm^{-1})	**Grain Size** (nm)
0	1.92	3.27	0.159	35.5
0.33	1.92	3.27	0.147	38.5
0.66	1.92	3.27	0.143	39.6
1	1.92	3.27	0.140	40.5
2	1.92	3.26	0.134	42.3
3	1.93	3.26	0.131	43.2
4	1.93	3.26	0.128	44.0
5	1.93	3.26	0.126	44.9
6	1.93	3.26	0.125	45.4
7	1.93	3.26	0.124	45.6
8	1.93	3.25	0.123	45.8
9	1.93	3.25	0.122	46.2
10	1.93	3.25	0.122	46.8
11	1.93	3.25	0.121	46.6
12	1.93	3.25	0.120	46.9
13	1.93	3.25	0.121	46.9
14	1.93	3.25	0.119	47.5
15	1.93	3.25	0.119	47.3
16	1.93	3.25	0.121	46.9
17	1.93	3.25	0.120	47.2
18	1.94	3.25	0.119	47.4
19	1.94	3.25	0.119	47.4
20	1.94	3.25	0.119	47.5
21	1.94	3.25	0.118	47.8
22	1.94	3.24	0.119	47.6
23	1.94	3.24	0.120	47.3
24	1.94	3.24	0.119	47.7

q^* is the scattering vector of the primary Bragg diffraction peak and the value obtained at the center of a Lorentzian distribution fit to each Bragg diffraction maximum is given. The period *L* is calculated as $2\pi/q$ using q^*. The full width at half maximum (FWHM) is taken from the parameters of the Lorentzian distribution giving the best fit. Grain sizes are calculated based on a Scherrer-analysis of the FWHM of the first order diffraction peak with $0.9 \times (2\pi/\text{FWHM})$.

Supplementary Table 7. Mechanical properties of TAB:Zn films with a varying processing history, *i.e.*, compression molded films that were treated at 220 °C (8 tons, 10 s) or 180 °C (8 tons, 30 s) and subsequently cooled at a rate of ca. 5 °C min^{-1}, and compression molded films (180 °C, 8 tons, 30 s) that were further annealed at 180 °C in an oven for 24 h. Data represent averages of n = 3-7 individual measurements ± standard deviation.

**TAB:Zn sample**	***E*' (25 °C)** (MPa)[a]	***E*' (170 °C)** (MPa)[a]	**Failure Temp.** (°C)[a]	**T_m** (°C)[b]	**ΔH_m** (J g^{-1})[b]	**Young's modulus** (GPa)[c]	**Tensile strength** (MPa)[c]	**Strain at break** (%)[c]
Compression-molded 220 °C / slow cooling	1419 ± 66	140 ± 3	215	212	6.1	1.11 ± 0.07	4.5 ± 0.3	0.41 ± 0.02
Compression-molded 180°C / slow cooling	1530 ± 51	252 ± 15	220	217	7.1	1.14 ± 0.04	4.2 ± 0.4	0.35 ± 0.03
Compression-molded 180 °C / 24 h	1522 ± 91	427 ± 24	230	224	8.2	1.11 ± 0.07	4.4 ± 0.5	0.37 ± 0.04

[a]Measured by DMA. [b]Measured by DSC. [c]Measured by stress-strain experiments at 25 °C with a strain rate of 1 % min^{-1}.

Supplementary Table 8. Data obtained by analysis of the SAXS/WAXS scattering profiles for BKB:Zn films with a varying processing history, *i.e.*, compression molded films (180 °C, 8 tons, 5 min) and films that were additionally annealed at a temperature of 180 °C in an oven for 24 h.

**BKB:Zn sample**	$q^{\#}$ (nm^{-1})	L (nm)	**FWHM** (nm^{-1})	**Grain Size** (nm)
Compression-molded	0.70	8.98	0.155	36.9
Compression-molded annealed 180° 24 h	0.71	8.85	0.061	92.7

q^* is the scattering vector of the primary Bragg diffraction peak and the value obtained at the center of a Lorentzian distribution fit to each Bragg diffraction maximum is given. The period L is calculated as $2\pi/q$ using q^*. The full width at half maximum (FWHM) is taken from the parameters of the Lorentzian distribution giving the best fit. Grain sizes are calculated based on a Scherrer-analysis of the FWHM of the first order diffraction peak with $0.9 \times (2\pi/\mathrm{FWHM})$.

Supplementary Table 9. Comparison of the data obtained by analysis of the small- and wide-angle X-ray scattering (SAXS/WAXS) profiles of the neat TAB:Zn and BKB:Zn metallosupramolecular polymers and the TAB/BKB:Zn copolymers with a TAB/BKB weight ratio of 80:20, 50:50, and 20:80 wt/wt% for samples that were compression-molded and additionally annealed for 24 h in an oven at a temperature of 180 °C.

**Composition**	q^* (nm^{-1})	L^* (nm)	$q^\#$ (nm^{-1})	$L^\#$ (nm)	q^0_{WAXS} (nm^{-1})	L^0 (Å)
**TAB:Zn**	–	–	1.98	3.17	6.36	9.9
**TAB/BKB:Zn (80/20)**	0.76	8.27	1.90	3.31	5.95	10.5
**TAB/BKB:Zn (50/50)**	0.78	8.06	1.90	3.31	5.96	10.5
**TAB/BKB:Zn (20/80)**	0.78	8.06	–	–	5.94	10.5
**BKB:Zn**	0.72	8.72	–	–	6.49	9.7

q^* and $q^\#$ are the scattering vectors of the primary Bragg diffraction peak of each pattern, and q^0_{WAXS} are the scattering vectors of the primary WAXS diffraction peak of each pattern. The values obtained at the maximum intensity of the primary Bragg diffraction peak to each Bragg diffraction maximum is given. The period L^*, $L^\#$, and L^0 are calculated as $2\pi/q$ using q^*, $q^\#$, or q^0_{WAXS}, respectively.

Supplementary Table 10. Mechanical properties of TAB:Zn films used to demonstrate optical healing. Data represent averages of n = 3–7 individual measurements ± standard deviation.

**Sample**	**Young's Modulus** (GPa)[a]	**Tensile strength** (MPa)[a]	**Strain at break** (%)[a]	**Toughness** (kJ·m^{-3})[a]
**Original**	1.11 ± 0.07	4.5 ± 0.3	0.4 ± 0.1	9.4 ± 0.9
**Damaged**[b]	1.01 ± 0.13	2.1 ± 0.07	0.2 ± 0.1	2.4 ± 1.6
**Healed**[c]	1.00 ± 0.02	4.5 ± 0.07	0.5 ± 0.1	10.4 ± 0.5

[a]Measured by stress-strain experiments at 25 °C with a strain rate of 1 %· min^{-1}. [b]Samples were damaged in a controlled manner by cutting to a depth of ~30% of their original thickness. [c]Samples were optically healed by exposure to light of a wavelength of 320–390 nm and a power density of 320 mW·cm^{-2} for 12 s.

Supplementary Table 11. Mechanical properties of the TAB/BKB:Zn copolymer containing TAB and BKB in a 50:50 (wt/wt%) ratio used to demonstrate thermally-induced healing. Data represent averages of n = 3–4 individual measurements ± standard deviation.

**Sample**	**Young's Modulus** (MPa)[a]	**Tensile strength** (MPa)[a]	**Strain at break** (%)[a]	**Toughness** ($kJ\cdot m^{-3}$)[a]
**Original**	395 ± 49	11.6 ± 0.5	10.4 ± 0.4	94 ± 4
**Damaged**[b]	336 ± 13	7.0 ± 1.2	3.2 ± 0.5	14 ± 6
**Healed**[c]	308 ± 39	9.9 ± 0.6	11.0 ± 1.1	87 ± 13

[a]Measured by stress-strain experiments at 25 °C with a strain rate of 1 %· min^{-1}. [b]Samples were damaged in a controlled manner by cutting to a depth of ~30% of their original thickness. [c]Samples were thermally healed at temperatures of 220°C for 45 min.

3. Supplementary Methods 1

General Synthesis Procedures and Reagents. Unless otherwise noted, all reactions were carried out in dried Schlenk glassware in an inert nitrogen atmosphere. Spectroscopy grade $CHCl_3$ was purchased from Acros and purified from acidic impurities prior to use by passage through a plug of dry, activated (Brockman I) basic alumina. Hydroxyl-terminated poly(ethylene-*co*-butylene) (Krasol HLBH-P 3000; M_n = 3100 g mol^{-1}, polydispersity index = 1.05) was kindly donated by Cray Valley SA and used as received. 11-Bromo-1-undecene, 1,3,5-trihydroxybenzene, 9-borabicyclo[3.3.1]nonane (9-BBN), triphenyl-phosphine, diethyl azodicarboxylate, and activated charcoal were purchased from Sigma Aldrich and used as received. Zinc bis(trifluoromethylsulfonyl)imide (>97%, Strem Chemicals, Inc.), anhydrous CH_3CN (Acros), anhydrous DMF (Acros), anhydrous THF (Acros), Keyplast FL Yellow R (Milliken & Company), Keyplast FL Red KB (Milliken & Company), and all other reagents and solvents (Sigma Aldrich) were used as received without further purification. The 4-hydroxy-2,6-bis(*N*-methylbenzimidazol-2'-yl)pyridine (Mebip), telechelic bis(2,6-bis(*N*-methylbenzimidazol-2'-yl)pyridine)-functionalized poly(ethylene-*co*-butylene) (BKB) macromonomer with a number-average molecular weight (M_n) of 4400 g mol^{-1}, and the neat metallosupramolecular polymer BKB:Zn were synthesized as previously reported.[3,4] Thin layer chromatography (TLC) was performed on TLC plates from Merck (Silica gel 60 F254). UV-light (254 nm), potassium permanganate staining, or Seebach's staining agent[5] were used for detection. Column chromatography was conducted on Geduran silica gel Si 60 from Merck (40-60 μm).

Chemical Analytics. ^{1}H (400 MHz) and ^{13}C (100 MHz) NMR spectra were recorded on a Bruker AVIII HD spectrometer in either $CDCl_3$, DMSO-d_6, or CD_3CN. Spectra were calibrated to the residual solvent peak of $CDCl_3$ (7.26 ppm ^{1}H NMR; 77.16 ppm ^{13}C NMR), DMSO-*d*6 (2.50 ppm ^{1}H NMR; 39.52 ppm ^{13}C NMR), or CD_3CN (1.94 ppm ^{1}H NMR). Data were evaluated with the MestReNova software suite (v 12.0) and all chemical shifts δ are reported in parts per million (ppm) relative to tetramethylsilane (TMS) with coupling constant in Hz (multiplicity: s = singlet, d = doublet, dd = double doublet, t = triplet, m = multiplet, br = broad signal). High resolution (HR) electrospray ionization mass spectrometry (ESI-MS) data were

acquired on a Bruker maXis Q-TOF instrument operated in positive ionization mode. Spectra were calibrated internally using Agilent ESI L low concentration tuning mix (AGG1969-85000).

Microscopy. Optical microscopy images were acquired on an Olympus BX51 microscope equipped with a DP72 digital camera. For temperature-dependent microscopy experiments, a Linkam LTS 350 heating stage was mounted to the microscopy. Experiments were carried out with heating and cooling rates of 10 °C min^{-1}.

Shear Rheology. Rheological measurements were performed using a TA-Instruments ARES-G2 with a nitrogen gas stream for temperature control. A geometry with parallel plates with a diameter of 8 mm was used and the sample thickness was around 0.3 mm. The limit for linear response behavior was initially checked by strain sweep experiments at a fixed angular frequency. Afterwards, rheological measurements in the linear regime were conducted in a range of temperatures and angular frequencies. Measurements at each temperature were performed after an equilibration time of 5 min. Frequency sweep experiments (16 data points between ω = 0.1 and ω = 100 rad s^{-1}) were acquired between 80 and 200 °C in 20 °C steps as well as between 200 °C and 230 °C in 10 °C steps. For clarity, temperature-dependent data are reported for one frequency only.

Preparation of Welded or Compositionally Graded TAB/BKB:Zn Copolymer Films. Films of the TAB/BKB:Zn copolymers with TAB/BKB ratios 10:90 and 60:40 wt/wt% were prepared in a procedure that mirrors the general metallosupramolecular polymerization and film formation, but the samples were dyed to allow for an optical differentiation. In the case of TAB/BKB:Zn copolymers with a TAB/BKB ratio of 10:90 wt/wt% a yellow dye (Keyplast FL Yellow R) was used whereas for copolymers with a TAB/BKB ratio of 60:40 wt/wt% a red dye (Keyplast FL Red KB) was employed. The respective dye (ca. 1 mg) was added to the polymer solutions prior to solvent casting and the film formation was carried out as described above.

To weld two films in a lap joint geometry, rectangular films of the different copolymers with a thickness of ca. 200 µm, a length of ca. 1–1.5 cm, and a width of 5.5 mm were placed on a Kapton sheet so that the

ends of the films overlapped by ca. 2.5 mm. Aluminum spacers (thickness of ca. 200 µm) were placed around the films, the assembly was covered with another Kapton sheet, and samples were welded by compression molding in a Carver CE press at a temperature of 180 °C for 10 s with a pressure of 6 tons. The samples were cooled to room temperature between the metallic plates of the press that were placed on a bench, before they were, after ca. 30 min, removed from the plates and cut into rectangular-shaped samples with typical dimensions (length × width × thickness) of ca. 20 × 5.5 × 0.2 mm.

To prepare compositionally graded samples, rectangular films of the copolymers with TAB/BKB ratios of 10:90 and 60:40 wt/wt% with thicknesses of ca. 200 µm, lengths of ca. 1–1.5 cm, and a width of 5.5 mm were cut diagonally into two pieces. Two pieces, one of each composition, were placed on a Kapton sheet and aligned with the cut interfaces facing each other so that a rectangular-shaped sample composed of the different copolymers was obtained (see graphical guide below). Aluminum spacers (thickness of ca. 200 µm) were placed around the films, the assembly was covered with another Kapton sheet, and samples were welded by compression molding in a Carver CE press at a temperature of 180 °C for 10 s with a pressure of 6 tons. After cooling was done as described above, the resulting film with a graded composition was cut into strips having a width of 1–2 mm, the individual strips were stacked on top of each other, sandwiched between glass slides and Kapton sheets, clamped, and welded at 120 °C for 3 h in an oven. The sample was removed, placed between Kapton sheets that were kept apart by 200 µm thick aluminum spacers, and compression molded in a Carver CE press at a temperature of 220 °C for 10 s with a pressure of 6 tons. The metallic plates of the press were placed on the bench and the samples were left to cool to room temperature between the plates over the course of ca. 45 min. The obtained film was cut into rectangular-shaped samples with typical dimensions (length × width × thickness) of ca. 15 × 5.5 × 0.2 mm.

Graphical Guide for the Preparation of Compositionally Graded TAB/BKB:Zn Copolymer Films:

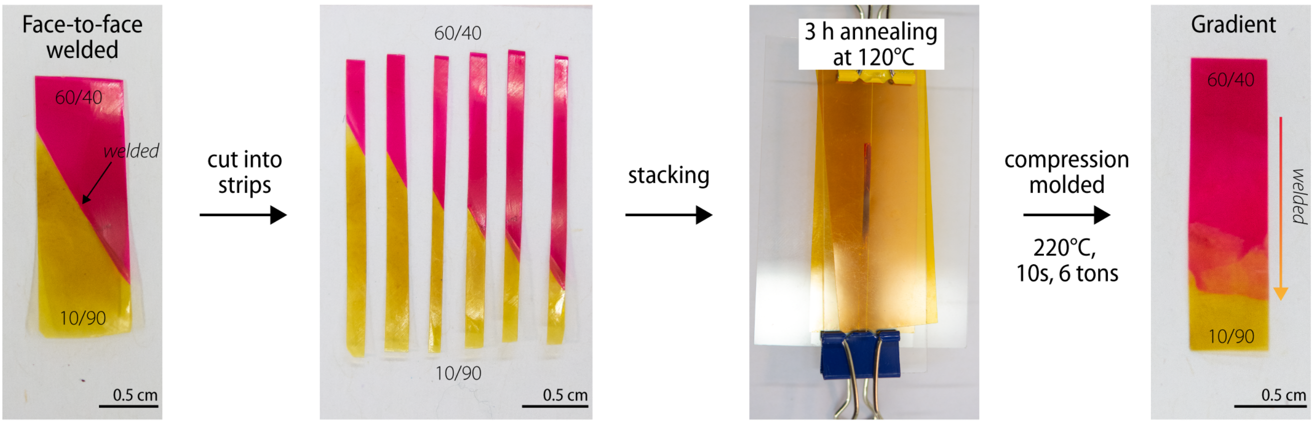

From left: two diagonally cut pieces of the copolymers with TAB/BKB ratios of 10:90 (yellow) and 60:40 wt/wt% (red) were aligned and welded. The film was cut into strips. Individual strips were stacked, sandwiched between glass slides and Kapton sheets, clamped, and welded. Thin films with a graded composition are obtained by compression molding.

4. Supplementary Methods 2

Synthetic Procedures and Analytical Data

1,3,5-Tris(undec-10-en-1-yloxy)benzene 1. Potassium carbonate (11.0 g, 79.2 mmol) and 11-bromo-1-undecene (6.1 g; 26.1 mmol) were added to a stirred solution of 1,3,5-trihydroxybenzene (1.0 g, 7.92 mmol) in dry DMF (38 mL). The resulting suspension was heated to 110 °C and stirred for 24 h. The reaction mixture was cooled to room temperature, filtered, and the solvent was removed *in vacuo*. The solid residue was dissolved in EtOAc (20 mL) and water was added (20 mL), the organic phase was separated, the aqueous layer was extracted twice with EtOAc (2 × 20 mL), the combined organic phases were dried over Na_2SO_4, and filtered. After removal of the solvent *in vacuo*, the crude mixture was purified by column chromatography (silica gel; DCM:hexane 1:4 to 2:3 v/v) to obtain **1** as a colorless liquid (1.8 g, 3.08 mmol, 40%).

^{1}H NMR (400 MHz, $CDCl_3$): δ = 6.06 (s, 3H, 3 –C$H_{arom.}$), 5.82 (ddt, 3H, J = 17, 10, 7 Hz, 3 CH=CH_2), 4.96 (m, 6H, 3 CH=CH_2), 3.90 (t, 6H, J = 7 Hz, 3 –CH_2CH_2O–), 2.05 (m, 6H, 3 CH_2CH=CH_2), 1.87-1.64 (m, 6H, 3 –C$H_2$$CH_2$O–), 1.51-1.22 (m, 36H, 3 –CH_2(CH_2)$_6$$CH_2$–). ^{13}C NMR (100 MHz, $CDCl_3$): δ = 161.1, 139.4, 114.3, 93.9, 68.1, 34.0, 29.7, 29.6, 29.5, 29.4, 29.3, 29.1, 26.2. HRMS (m/z; ESI pos. mode): calcd. for $C_{39}H_{67}O_3$: 583.5085 ([M+H]$^+$); found: 583.5080.

1,3,5-Tris(11-hydroxyundecyloxy)benzene 2. A solution of **1** (1.50 g, 2.57 mmol) in dry THF (10 mL) was added dropwise over the course of 10 min to a solution of 9-borabicyclo[3.3.1]nonane (9-BBN) (18.5 mL, 0.5 M, 9.26 mmol) in THF that was kept in an ice bath and maintained at 0 °C. After the addition was complete, the cooling bath was removed, and the reaction mixture was stirred for 8 h at room temperature. Water (1.4 mL) was then added and the reaction mixture was cooled to 0 °C. Aqueous sodium hydroxide (6.8 mL, 3 M) and hydrogen peroxide (6.8 mL, 30%) solutions were slowly added. The cooling bath was removed after the addition was complete and the reaction mixture was stirred for 12 h at room temperature. The mixture was poured into a mixture of DCM (50 mL) and dilute aqueous HCl (5%, 50 mL). The organic phase was separated off, washed with brine, dried over Na_2SO_4, and filtered. The filtrate

was concentrated *in vacuo* and purification by column chromatography (silica gel; DCM/MeOH 10:1 v/v) afforded **2** as a colorless solid (1.42 g, 2.23 mmol, 87 %).

^{1}H NMR (400 MHz, $CDCl_3$): δ = 6.05 (s, 3H, 3 –C$H_{arom.}$), 3.89 (t, 6H, J = 7 Hz, 3 –CH_2CH_2O–), 3.63 (t, 6H, J = 7 Hz, 3 –CH_2OH), 1.74 (quin, 6H, J = 7 Hz, 3 –CH_2CH_2O–), 1.56 (quin, 6H, J = 7 Hz, 3 –CH_2CH_2OH), 1.47-1.24 (m, 45H, 3 –$CH_2(CH_2)_7CH_2$– / 3 –CH_2OH). ^{13}C NMR (100 MHz, $CDCl_3$): δ = 161.1, 94.0, 68.2, 63.2, 33.0, 29.7, 29.7, 29.6, 29.6, 29.5, 29.4, 26.2, 25.9. HRMS (m/z; ESI pos. mode): calcd. for $C_{39}H_{73}O_6$: 637.5402 ([M+H]$^+$); found: 637.5400.

1,3,5-Tris((11-((2,6-bis(1-methyl-1*H*-benzo[*d*]imidazol-2-yl)pyridin-4-yl)oxy)undecyl)oxy)benzene (TAB). 4-Hydroxy-2,6-bis(*N*-methylbenzimidazol-2'-yl)pyridine[3] (1.07 g, 3.02 mmol), triphenylphosphine (1.58 g, 6.04 mmol), and **2** (0.40 g, 0.63 mmol) were dissolved in dry THF (20 mL) and the solution was cooled to −40 °C. A solution of diethyl azodicarboxylate (3.2 mL, 40 wt%) in toluene was added, the reaction mixture was stirred at –40 °C for 4 h, the cooling bath was removed, stirring was continued for 48 h, and the solvents were removed *in vacuo*. The residue was dissolved in DCM (ca. 5 mL) and precipitated into diethyl ether that had been cooled to ca. –20 °C in the freezer (150 mL). The precipitation was repeated once, the solid residue was dissolved in in $CHCl_3$ (50 mL), activated charcoal (ca. 1 g, powder, mesh ~100) was added, and the mixture was stirred for 1 h. The mixture was filtered through a pad of Celite® S, the solvent was removed *in vacuo*, the solid was dissolved in DCM (ca. 5 mL) and precipitated into cold (ca. –20 °C) diethyl ether (150 mL). The precipitate was dried *in vacuo* over night at 50 °C to obtain the title compound as a pinkish solid (633 mg, 0.38 mmol, 61%).

^{1}H NMR (400 MHz, $CDCl_3$): δ = 7.94 (s, 6H, 6 –C$H_{arom.}$), 7.88-7.86 (m, 6H, 6 –C$H_{arom.}$), 7.46-7.44 (m, 6H, 6 –C$H_{arom.}$), 7.40-7.32 (m, 12H, 12 –C$H_{arom.}$), 6.05 (s, 3H, 6 –C$H_{arom.}$), 4.25-4.22 (m, 24H, 6 –NCH_3 / 3 –CH_2CH_2OMebip), 3.90-3.87 (m, 6H, 3 –CH_2CH_2O–), 1.88-1.81 (m, 6H, 3 –CH_2CH_2O–), 1.78-1.71 (m, 6H, 3 –CH_2CH_2OMebip) 1.48-1.25 (m, 42H, 3 –$CH_2(CH_2)_7CH_2$–). ^{13}C NMR (100 MHz, $CDCl_3$): δ = 166.9, 161.1, 151.1, 150.5, 142.5, 137.3, 123.7, 123.0, 120.2, 112.1, 110.1, 93.9, 68.9, 68.2, 32.7, 29.7, 29.5, 29.4, 29.0, 26.2, 26.0. HRMS (m/z; ESI pos. mode): calcd. for $C_{102}H_{118}N_{15}O_6$: 1648.9384 ([M+H]$^+$); found: 1648.9368.

Bis-(2,6-bis(*N*-methylbenzimidazol-2'-yl)pyridine)-functionalized telechelic poly(ethylene-*co*-butylene) (BKB). The preparation of the **BKB** macromonomer was carried out following previously reported synthetic procedures.[3,4]

^{1}H NMR (400 MHz, $CDCl_3$): δ = 7.97 (s), 7.89 (d), 7.48-7.34 (m), 4.29–4.24 (m), 2.00–0.89 (m), 0.85–0.65 (m). ^{13}C NMR (100 MHz, $CDCl_3$): δ = 166.9, 151.2, 150.4, 137.2, 123.8, 123.1, 120.2, 112.1, 110.1, 69.0, 39.2–38.6, 36.3, 33.7, 30.8, 30.4, 29.9, 27.0, 26.7, 26.3, 26.2, 11.0, 10.8, 10.6.

5. Supplementary Methods 3

NMR Spectra

^{1}H NMR spectrum ($CDCl_3$, 400 MHz) of **1**.

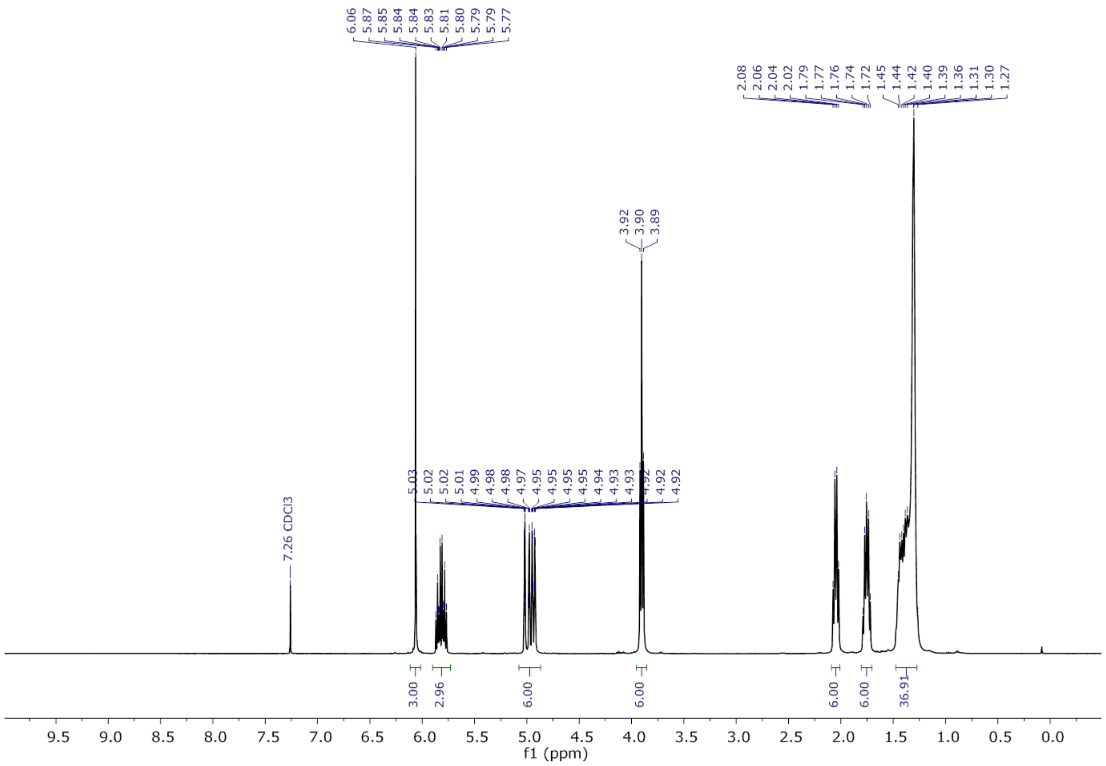

^{13}C NMR spectrum ($CDCl_3$, 100 MHz) of **1**.

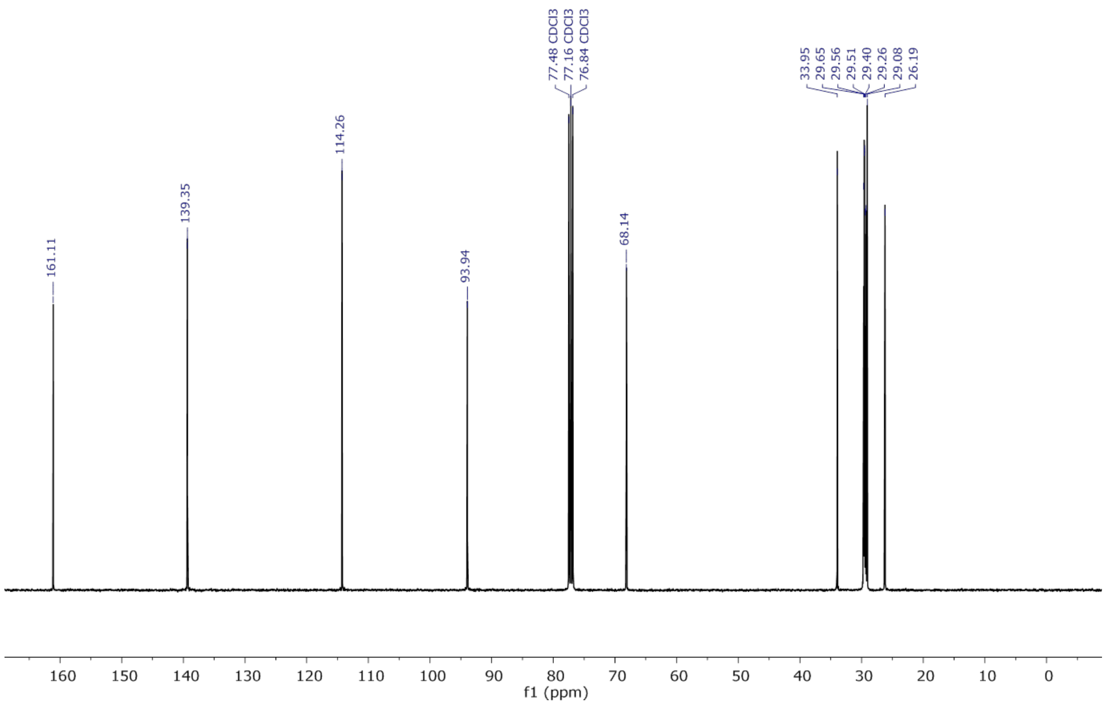

^{1}H NMR spectrum ($CDCl_3$, 400 MHz) of **2**.

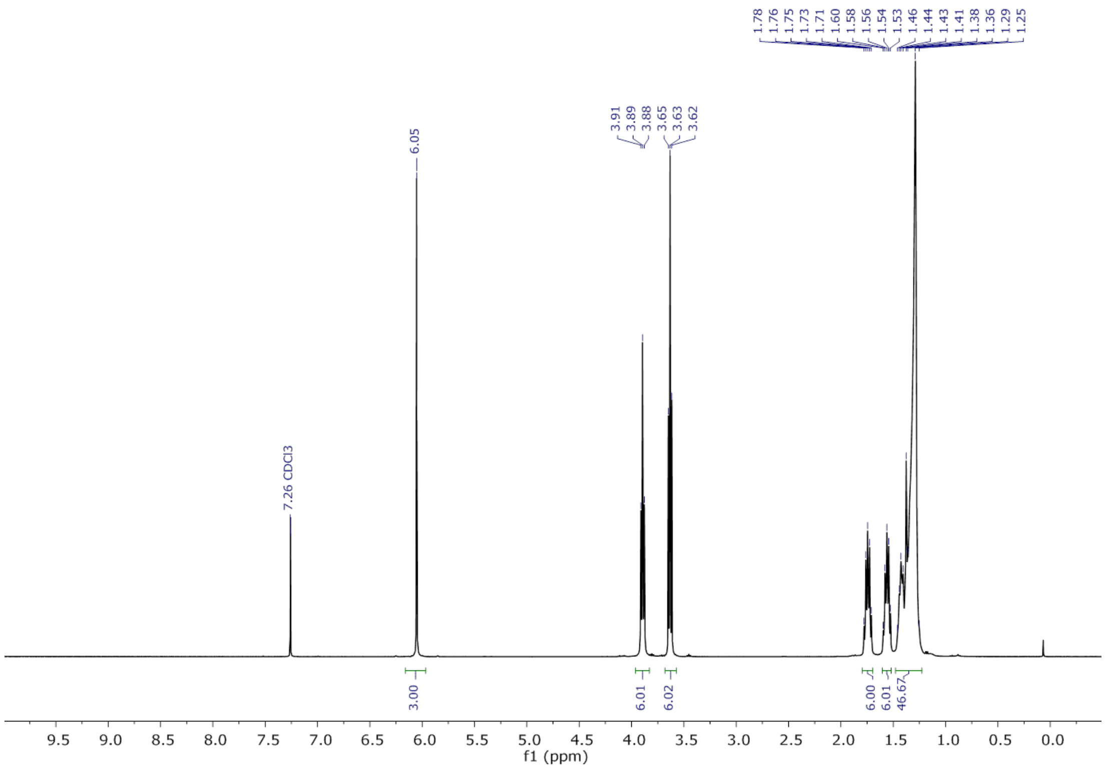

^{13}C NMR spectrum ($CDCl_3$, 100 MHz) of **2**.

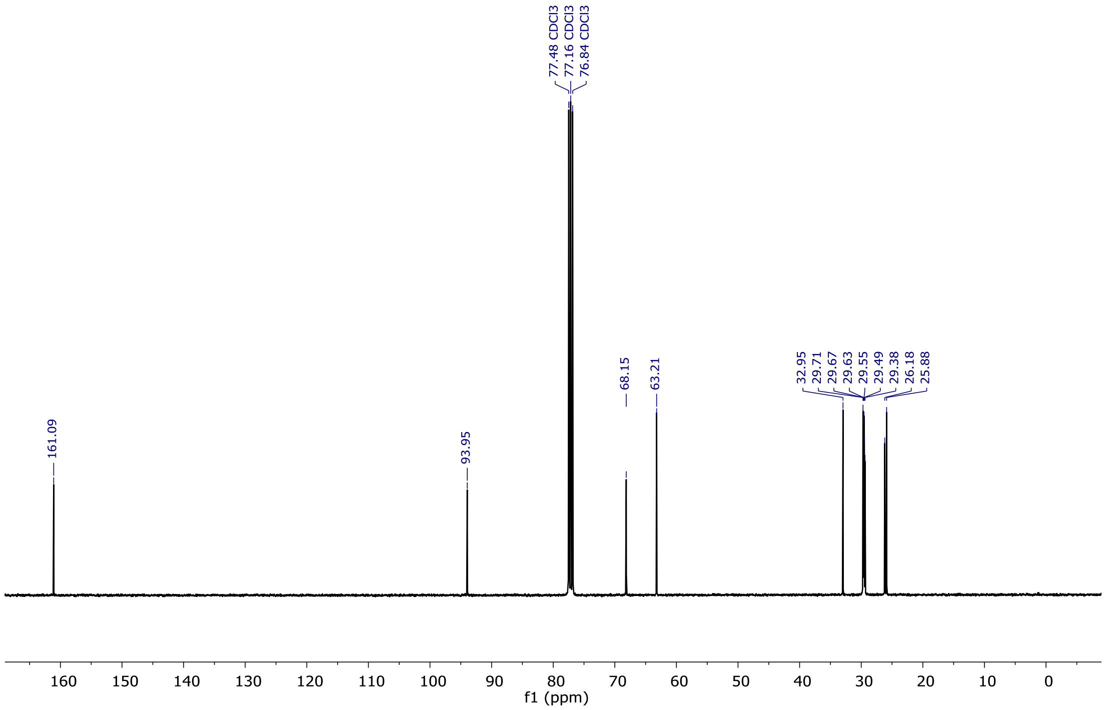

^{1}H NMR spectrum ($CDCl_3$, 400 MHz) of **TAB**.

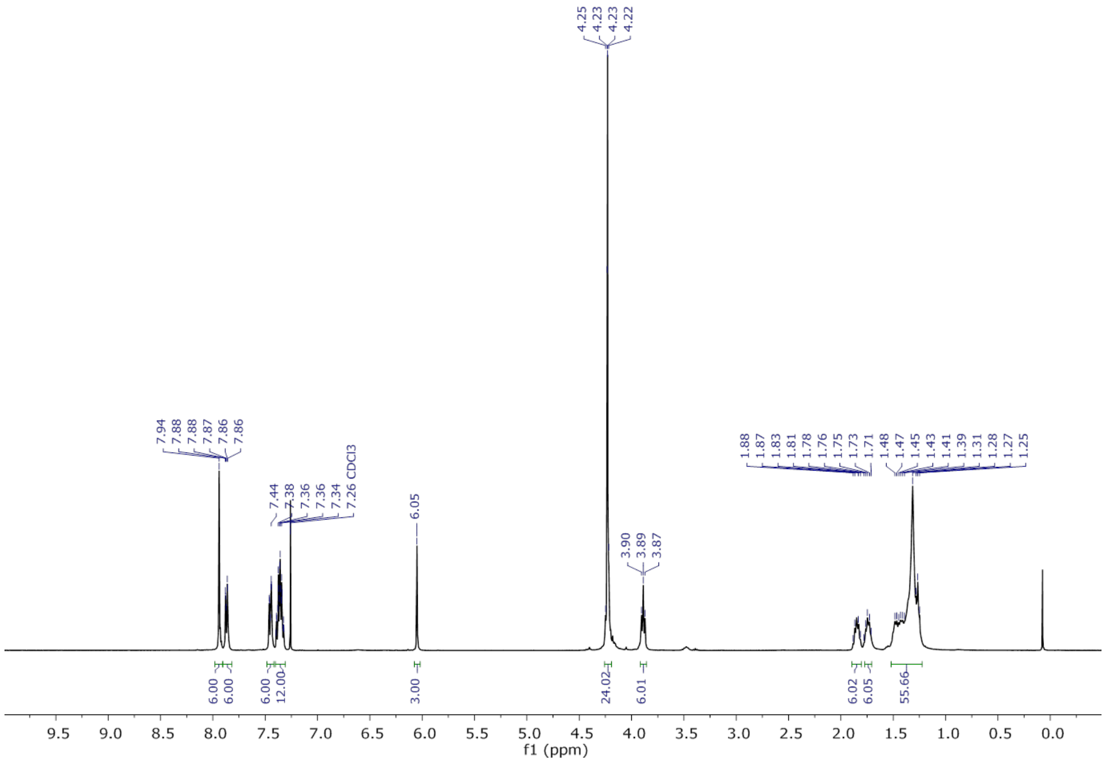

^{13}C NMR spectrum ($CDCl_3$, 100 MHz) of **TAB**.

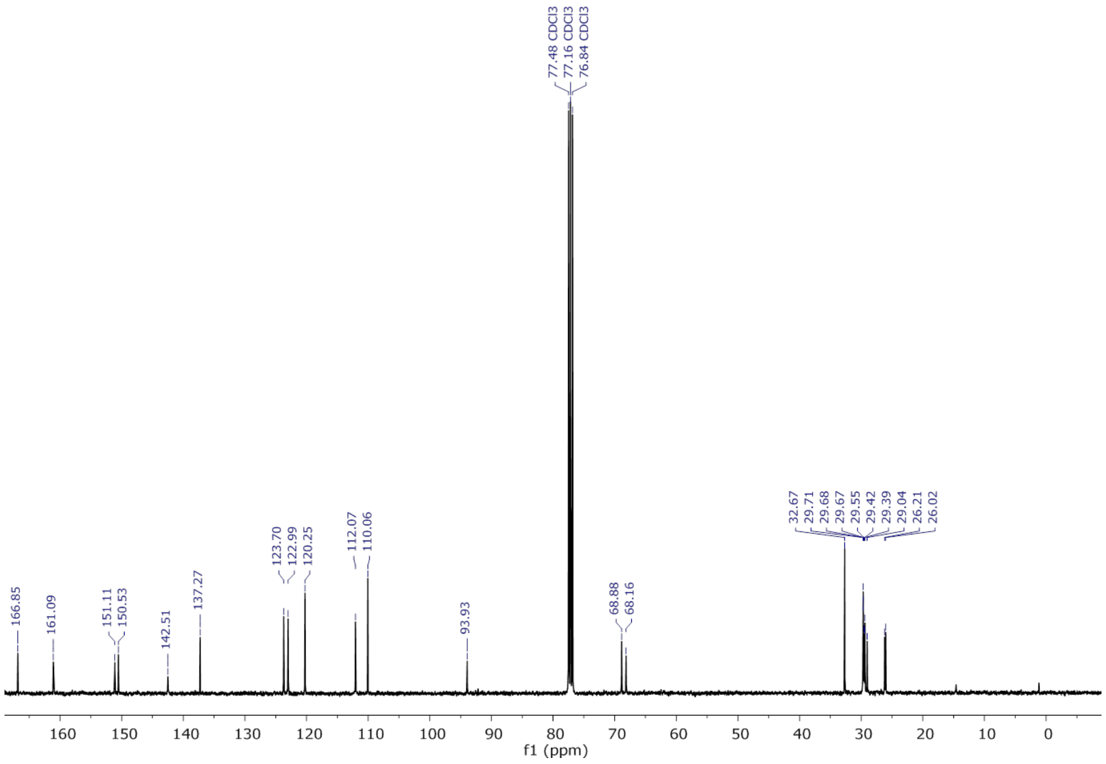

6. Supplementary References

1. Ashby, M. F. Materials selection in mechanical design. (Butterworth-Heinemann, 2011). doi:10.1016/C2009-0-25539-5.

2. Wypych, G. Handbook of polymers. (ChemTech Publishing, 2012). doi:10.1016/C2011-0-04631-8.

3. Rowan, S. J. & Beck, J. B. Metal–ligand induced supramolecular polymerization: A route to responsive materials. *Faraday Discuss.* **128**, 43–53 (2005).

4. Burnworth, M. et al. Optically healable supramolecular polymers. *Nature* **472**, 334–337 (2011).

5. Seebach, D., Imwinkelried, R. & Stucky, G. Optisch aktive Alkohole aus 1,3-Dioxan-4-onen: eine praktikable Variante der enantioselektiven Synthese unter nucleophiler Substitution an Acetal-Zentren. *Helv. Chim. Acta* **70**, 448–464 (1987).
