# Supplementary figures and images for: Mechanically robust supramolecular polymer co-assemblies
Source: Nat Commun. 2022 Jan 18;13:356. doi: 10.1038/s41467-022-28017-0 (PMC8766479; doi:10.1038/s41467-022-28017-0)

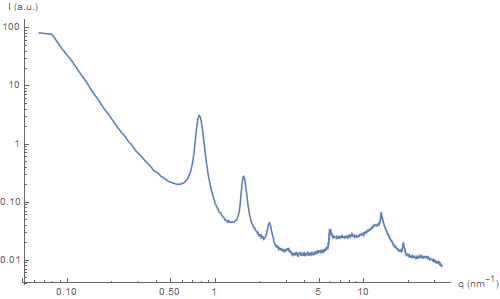

Supplement: Supplementary file 3 — Source Data [file 41467_2022_28017_MOESM3_ESM.zip › NCOMMS-21-41006-T_Source Data/Supplementary Figure 26/annealed 2080/2080_code_0mg_id_iq.tiff]

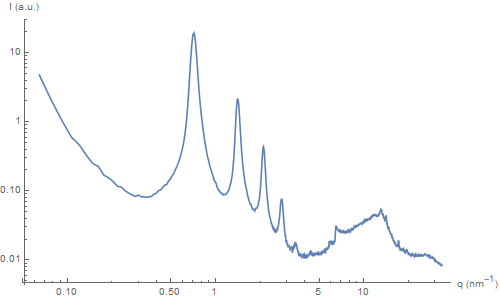

Supplement: Supplementary file 3 — Source Data [file 41467_2022_28017_MOESM3_ESM.zip › NCOMMS-21-41006-T_Source Data/Supplementary Figure 26/annealed BKBZn/BKB_code_0mg_id_iq.tiff]

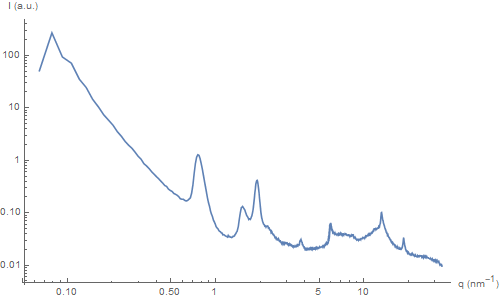

Supplement: Supplementary file 3 — Source Data [file 41467_2022_28017_MOESM3_ESM.zip › NCOMMS-21-41006-T_Source Data/Supplementary Figure 26/annealed 8020/8020_code_0mg_id_iq.tiff]

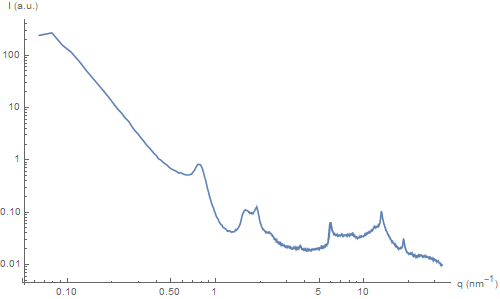

Supplement: Supplementary file 3 — Source Data [file 41467_2022_28017_MOESM3_ESM.zip › NCOMMS-21-41006-T_Source Data/Supplementary Figure 26/annealed 5050/5050_code_0mg_id_iq.tiff]
